# In vitro and in vivo studies of triacetone triperoxide (TATP) metabolism in humans

**DOI:** 10.1007/s11419-020-00540-z

**Published:** 2020-07-14

**Authors:** Michelle D. Gonsalves, Kevin Colizza, James L. Smith, Jimmie C. Oxley

**Affiliations:** Chemistry Department, University of Rhode Island, 140 Flagg Rd, Kingston, RI 02881 USA

**Keywords:** Triacetone triperoxide (TATP), Terrorists, Human in vitro and in vivo metabolism for TATP exposure, TATP-*O*-glucuronide, CYP2B6 hydroxylation, UGT2B7 glucuronidation

## Abstract

**Purpose:**

Triacetone triperoxide (TATP) is a volatile but powerful explosive that appeals to terrorists due to its ease of synthesis from household items. For this reason, bomb squad, canine (K9) units, and scientists must work with this material to mitigate this threat. However, no information on the metabolism of TATP is available.

**Methods:**

In vitro experiments using human liver microsomes and recombinant enzymes were performed on TATP and TATP-OH for metabolite identification and enzyme phenotyping. Enzyme kinetics for TATP hydroxylation were also investigated. Urine from laboratory personnel collected before and after working with TATP was analyzed for TATP and its metabolites.

**Results:**

While experiments with flavin monooxygenases were inconclusive, those with recombinant cytochrome P450s (CYPs) strongly suggested that CYP2B6 was the principle enzyme responsible for TATP hydroxylation. TATP-*O*-glucuronide was also identified and incubations with recombinant uridine diphosphoglucuronosyltransferases (UGTs) indicated that UGT2B7 catalyzes this reaction. Michaelis–Menten kinetics were determined for TATP hydroxylation, with *K*_m_ = 1.4 µM and *V*_max_ = 8.7 nmol/min/nmol CYP2B6_._ TATP-*O*-glucuronide was present in the urine of all three volunteers after being exposed to TATP vapors showing good in vivo correlation to in vitro data. TATP and TATP-OH were not observed.

**Conclusions:**

Since scientists working to characterize and detect TATP to prevent terrorist attacks are constantly exposed to this volatile compound, attention should be paid to its metabolism. This paper is the first to elucidate some exposure, metabolism and excretion of TATP in humans and to identify a marker of TATP exposure, TATP-*O*-glucuronide in urine.

**Electronic supplementary material:**

The online version of this article (10.1007/s11419-020-00540-z) contains supplementary material, which is available to authorized users.

## Introduction

Triacetone triperoxide (3,3,6,6,9,9-hexamethyl-1,2,4,5,7,8-hexoxonane, TATP) is a homemade explosive, easily synthesized from household items [1]. For this reason, TATP has often been used by terrorists [2, 3], necessitating its research by bomb squad, canine (K9) units, and scientists [4]. In addition to being extremely hazardous, this peroxide explosive is highly volatile, with partial pressure of 4–7 Pa at 20 ℃ [5, 6]. Personnel exposed to TATP will most likely absorb it through inhalation and/or dermal absorption. However, no information on the human absorption, distribution, metabolism, excretion and toxicity (ADMET) of TATP is available. Therefore, this paper will investigate the in vitro metabolism of TATP and the in vivo excretion through urine analysis.

The toxicity of most military explosives has been well characterized [7]. The biotransformation of trinitrotoluene, for example, has been thoroughly investigated. It is metabolized by cytochrome P450 (CYP) reductase, forming nitroso intermediates, and yielding 4-hydroxylamino-2,6-dinitrotoluene, 4-amino-2,6-dinitrotoluene and 2-amino-4,6-dinitrotoluene. These primary metabolites are further reduced by CYP to 2,4-diamino-6-nitrotoluene and 2,6-diamino-4-nitrotoluene [8, 9]. In vivo studies of Chinese ammunition factory workers found metabolites, such as 4-amino-2,6-dinitrotoluene and 2-amino-4,6-dinitrotoluene, in urine and bound to the hemoglobin in blood [9, 10]. TATP has been studied for almost two decades, but its metabolism and toxicity are still unknown. TATP characterization is problematic since it is an extremely sensitive explosive, difficult to handle and, due to its high volatility, difficult to concentrate in biological samples [5].

Most xenobiotics are metabolized by CYP which is a family of heme-containing enzymes found in all tissues, particularly the liver endoplasmic reticulum (microsomes). CYPs catalyze phase I oxidative reactions (among others) in the presence of oxygen and a reducing agent (usually reduced nicotinamide adenine dinucleotide phosphate, NADPH). NADPH provides electrons to the CYP heme via CYP reductase. This oxidation generally produces more polar metabolites that are either excreted in the urine or undergo phase II biotransformation, further increasing their hydrophilicity [11]. One of the most common phase II reactions is glucuronidation, which is catalyzed by uridine diphosphoglucuronosyltransferase (UGT) in the presence of the cofactor uridine diphosphoglucuronic acid (UDPGA). In this reaction, glucuronic acid is conjugated onto an electron-rich nucleophilic heteroatom, frequently added to the substrate by phase I metabolism. Glucuronide metabolites increase the topological polar surface area (TPSA) and reduce the partition coefficient (LogP) of xenobiotics to be ionized at physiological pH, thus, increasing the aqueous solubility of the compound for excretion [11].

TATP is a cyclic peroxide, a motif shared with the antimalarial drug, artemisinin. The endoperoxide functionality of artemisinin is thought to be crucial for its antimalarial activity [12]. In the presence of ferrous ions, artemisinin undergoes homolytic peroxide cleavage to yield an oxygen radical that may be lethal to malaria parasite, *Plasmodium falciparum*. Biotransformation studies indicate that artemisinin is primarily metabolized by CYP2B6 to deoxyartemisinin, deoxydihydroartemisinin, dihydroartemisinin and ‘crystal-7’ [13, 14]. Similarly, we have previously shown that TATP is metabolized in vitro by canine CYP2B11, another CYP2B subfamily enzyme [15]. Artemisinin is further metabolized by glucuronidation, particularly by UGT1A9 and UGT2B7, to dihydroartemisinin-glucuronide, the principal metabolite found in urine, suggesting endoperoxides, like TATP, may be glucuronidated and excreted in urine [16].

Laboratory personnel who work on synthesizing, characterizing and detecting TATP are inevitably exposed to this volatile compound. Even the small-sized samples that they work with can result in buildup of TATP in a confined space. Furthermore, bomb-sniffing dogs and their handlers are purposely exposed to these vapors for the sake of training. Our previous study revealed TATP metabolism in dog liver microsomes (DLM) [15]. Now we evaluate its in vitro biotransformation in human liver microsomes (HLM) and recombinant enzymes, identifying phase I and phase II metabolites, estimating enzyme kinetics and also detecting urinary in vivo metabolites excreted from scientists exposed to TATP in their work environment.

## Materials and methods

### Chemicals

Optima HPLC grade methanol, Optima HPLC grade water, Optima HPLC grade acetonitrile, American Chemical Society (ACS) grade acetone, ACS grade methanol, ACS grade pentane, hydrochloric acid, ammonium acetate, dipotassium phosphate, monopotassium phosphate, magnesium chloride (MgCl_2_) and reduced glutathione (GSH) were purchased from Fisher Chemical (Fair Lawn, NJ, USA); NADPH, 1-aminobenzotriazole, methimazole, 1-naphthol and hydroxyacetone from Acros Organics (Morris Plain, NJ, USA); UDPGA, saccharolactone and 2,4-dichlorophenoxyacetic acid from Sigma-Aldrich (St. Louis, MO, USA); bupropion, benzydamine and alamethicin from Alfa Aesar (Ward Hill, MA, USA); oxcarbazepine from European Pharmacopoeia Reference Standard (Strasbourg, France); ticlopidine from Tokyo Chemical Industry (Tokyo, Japan); hydroxybupropion from Cerilliant Corporation (Round Rock, Texas, USA); deuterated acetone (acetone-*d*_6_) from Cambridge Isotope Labs (Cambridge, MA, USA); hydrogen peroxide (50%) from Univar (Redmond, WA, USA); HLM, rat liver microsomes (RLM), DLM and human lung microsomes (HLungM) from Sekisui XenoTech (Kansas City, KS, USA); human recombinant CYP (rCYP) bactosomes expressed in *Escherichia coli* (*E. coli*) from Cypex (Dundee, Scotland); human recombinant flavin monooxygenase (rFMO) supersomes and human recombinant UGT (rUGT) supersomes expressed in insect cells from Corning (Woburn, MA, USA).

### TATP, deuterated TATP (TATP-*d*_18_) and hydroxy-TATP (TATP-OH) synthesis

TATP was synthesized following the literature methods using hydrochloric acid as the catalyst [1]. TATP was purified by recrystallization, first with methanol/water (80:20, v/v) and then with pentane. TATP-*d*_18_ was synthesized as above using acetone-*d*_6_. TATP-OH was synthesized as above using hydrogen peroxide (50 wt%)/acetone/hydroxyacetone (2:1:1, v/v/v) [15]. TATP-OH was purified using a CombiFlash RF + system with an attached PurIon S MS system (Teledyne Isco, Lincoln, NE, USA), followed by two cycles of drying and reconstituting in solvent to sublime away the TATP. Separation was performed using a C-18 cartridge combined with a liquid chromatograph flow of 18 mL/min with 10% methanol (A) and 90% aqueous 10 mM ammonium acetate (B) for 1 min, before ramping to 35%A/65%B over 1 min, followed by another ramp to 95%A/5%B over the next 1 min, holding for 2 min, before a 30 s transition to initial conditions, with a hold of 2 min [15].

### Instrumental analyses 

Metabolite identification was performed by high‐performance liquid chromatography coupled to Thermo Scientific Exactive or Thermo Scientific LTQ Orbitrap XL high-resolution mass spectrometers (HPLC–HRMS) (Thermo Fisher Scientific, Waltham, MA, USA). A CTC Analytics PAL autosampler (CTC Analytics, Zwingen, Switzerland) was used for LC injections, solvent delivery was performed using a Thermo Scientific Accela 1200 quaternary pump, and data collection/analysis was done using Xcalibur software (Thermo Scientific, version 2.1).

Metabolite quantification was performed by high‐performance liquid chromatography coupled to AB Sciex Q-Trap 5500 triple quadrupole mass spectrometer (HPLC–MS/MS) (AB Sciex, Toronto, Canada). A CTC Analytics PAL autosampler was used for LC injections, solvent delivery was performed using a Thermo Scientific Accela 1200 quaternary pump and data collection/analysis was done with Analyst software (AB Sciex, version 1.6.2).

The HPLC method for all TATP derivatives was as follows: sample of 40 µL (Exactive and LTQ Orbitrap XL) or 20 µL (Q-Trap 5500) in acetonitrile/water (50:50, v/v) was injected into LC flow at 250 µL/min of 10%A/90%B for introduction onto a Thermo Syncronis C18 column (50 × 2.1 mm i.d., particle size 5 µm). Initial conditions were held for 1 min before ramping to 35%A/65%B over 1 min, followed by another ramp to 95%A/5%B over the next 1 min. This ratio was held for 2 min before reverting to initial conditions over 30 s, which was held for additional 2 min. The Exactive MS tune conditions for atmospheric pressure chemical ionization (APCI) in positive mode were as follows: N_2_ sheath gas flow rate, 30 arbitrary units (AU); N_2_ auxiliary gas flow rate, 30 AU; discharge current, 6 µA; capillary temperature, 220 ℃; capillary voltage, 25 V; tube lens voltage, 40 V; skimmer voltage, 14 V; and vaporizer temperature, 220 ℃. The Exactive MS tune conditions for electrospray ionization (ESI) in negative mode were as follows: N_2_ sheath gas flow rate, 30 AU; N_2_ auxiliary gas flow rate, 15 AU; spray voltage, − 3.4 kV; capillary temperature, 275 ℃; capillary voltage, − 35 V; tube lens voltage, − 150 V; and skimmer voltage, − 22 V. The LTQ Orbitrap XL MS tune conditions for ESI−, used for TATP-*O*-glucuronide verification, were as follows: N_2_ sheath gas flow rate, 30 AU; N_2_ auxiliary gas flow rate, 15 AU; spray voltage, − 4 kV; capillary temperature, 275 °C; capillary voltage, − 15 V; and tube lens voltage, − 84 V. The single-reaction monitoring settings were as follows: *m/z* 413.13 with isolation width *m/z* 1.7, activated by higher-energy collision dissociation at 35 eV. The Q-trap 5500 MS tune and multiple reaction monitoring (MRM) conditions are shown in Table 1. TATP and TATP-OH quantification was done as the area ratio to TATP-*d*_18_ (internal standard, IS) using a standard curve ranging 10–20,000 ng/mL and 10–500 (Fig. S1) or 500–8,000 ng/mL, respectively. TATP-*O*-glucuronide relative quantification was done as the area ratio to 2,4-dichlorophenoxyacetic acid (IS).

The HPLC method for bupropion, hydroxybupropion, benzydamine, benzydamine *N*-oxide, and oxcarbazepine (IS) was as follow: sample of 10 μL in acetonitrile/water (50:50, v/v) was injected into LC flow at 250 μL/min with 30%A/70%B for introduction onto a Thermo Scientific Acclaim Polar Advantage II C18 column (50 × 2.1 mm i.d., particle size 3 µm). Initial conditions were held for 1 min before instant increase to 95%A/5%B, held for 2.5 min, and then reversed to initial conditions over 30 s, with a hold of 1 min for the bupropion, hydroxybupropion and oxcarbazepine method or with a hold of 3 min for the benzydamine, benzydamine *N*-oxide and oxcarbazepine method.

**Table 1:** Triple quadrupole mass spectrometer (Q-trap 5500) operating parameters

Parameter	Method 1		Method 2	Method 3			
Source type	APCI +		ESI −	ESI +			
Source temperature (℃)	300		300	260			
Ion spray voltage (V)	N/A		− 4500	4500			
Nebulizer current (µA)	0.8		N/A	N/A			
Ion source gas 1 (psi)	50		50	20			
Ion source gas 2 (psi)	2		2	2			
Curtain gas (psi)	28		28	30			
Collision gas (psi)	6		6	5			
Declustering potential (V)	26		− 26	30			
Entrance potential (V)	10		− 10	10			
Internal standard MRM transitions (*m/z*)	258 → 80, 46		219 → 161, 125	253 → 208, 180			
Collision energy (V)	11, 27		− 13, − 27	27, 39			
Collision cell exit potential (V)	14, 20		− 36, − 20	22, 14			
Analyte	TATP	TATP-OH	TATP-*O*-gluc	BUP	BUP-OH	BZD	BZD-NO
Analyte MRM transition (*m/z*)	240 → 74, 43	256 → 75	413 → 113, 87	240 → 184, 166	256 → 238, 139	310 → 86	326 → 102
Collision energy (V)	11, 28	13	− 22, − 34	17, 23	15, 33	21	19
Collision cell exit potential (V)	10, 11	36	− 13, − 9	20, 10	12, 16	10	12

*APCI* + positive mode atmospheric pressure chemical ionization, *BUP* bupropion, *BUP-OH* hydroxybupropion, *BZD* benzydamine, *BZD-NO* benzydamine *N*-oxide, *ESI* + positive mode electrospray ionization, *ESI-* negative mode electrospray ionization, *MRM* multiple reaction monitoring, *N/A* not applicable, *TATP* triacetone triperoxide, *TATP-O-gluc* triacetone triperoxide-*O*-glucuronide, *TATP-OH* hydroxyl-triacetone triperoxide

### Metabolite identification

All incubations were performed in triplicate in a Thermo Scientific Digital Heating Shaking Drybath set to body temperature 37 ℃ and 800 rpm. An incubation mixture containing phosphate buffer (pH 7.4), MgCl_2_ [17], and NADPH (CYP cofactor [11]) was prepared so that at a final volume of 1 mL, their concentrations were 10, 2 and 1 mM, respectively. When the incubation times were relatively long (greater than 15 min), it was thought necessary to use closed vessels to avoid loss of the volatile TATP or TATP-OH; as a result, prior to incubation, oxygen gas was bubbled through the buffer to ensure ample oxygen availability [15]. To this mixture, microsomes or recombinant enzymes were added and equilibrated for 3 min before the reaction was initiated by adding the substrate. Substrates included TATP in acetonitrile, TATP-OH in methanol, and bupropion and benzydamine in water. Organic solvents can disrupt metabolism, but the catalytic activity of most CYP enzymes is unaffected by less than 1% acetonitrile or methanol [18]. At the end point, an aliquot was transferred to a vial containing equal volume of ice-cold acetonitrile and immediately vortex-mixed to quench the reaction. The sample was centrifuged for 5 min at 14,000 rpm, and the supernatant was analyzed by LC–MS.

Metabolite identification studies used microsomes (HLM, RLM and DLM) at protein concentrations of 1 mg/mL in the incubation mixture. The substrate, TATP, TATP-OH, or TATP-*d*_18_ (10 µg/mL) was allowed to incubate for several min before MS analysis. Negative controls consisted of the incubation mixture excluding either microsomes or NADPH. Positive control used 100 µM bupropion, a probe substrate for CYP2B6 [19–21].

Phase II TATP metabolism was examined by two studies. Metabolism by glutathione *S*-transferase (GST) was probed by equilibrating 5 mM GSH (GST cofactor [11]) for 5 min in the incubation mixture including HLM before the substrate (TATP) was added. Ticlopidine (10 µM) was the substrate for the GST positive control (Fig. S2) [22]. To examine metabolism by UGT, HLM, buffer, and alamethicin (50 µg/mL in methanol/water) were equilibrated cold for 15 min, before saccharolactone (1 mg/mL, *β*-glucuronidase inhibitor [23]), MgCl_2_, and NADPH were added, and the mixture was warmed to 37 ℃ and shaken at 800 rpm. After 3 min equilibration, the substrate (TATP or TATP-OH) was added, and in 2 min, the reaction was started by the addition of 5.5 mM UDPGA (UGT cofactor [11]) [24, 25]. Positive control used 100 µM 1-naphthol, an UGT1A6 substrate (Fig. S3) [26, 27]. Alamethicin was employed to replace membrane transporters, in allowing UGT (located in the endoplasmic reticulum lumen) easy access to the UDPGA cofactor [11].

### Enzyme identification

TATP was incubated as described in the previous section with various enzyme inhibitors in HLM (1 mg/mL). TATP-OH formation was first monitored and benchmarked against incubations without inhibitors. Chemical inhibitors, such as 1-aminobenzotriazole (1 mM) [28, 29], methimazole (500 µM) [30, 31], or ticlopidine (100 µM) [32, 33], were pre-equilibrated in the incubation mixture for 30 min prior to the addition of the substrate (100 µM, TATP or controls). TATP was also tested as a possible CYP2B6 inhibitor; TATP or bupropion was pre-equilibrated in the incubation mixture before starting the reaction with a known CYP2B6 substrate (bupropion) or TATP, respectively. FMO inhibition by heat was also tested [11, 34]. In that experiment, HLM was mixed with buffer and preheated at 37 or 45 ℃ for 5 min. After an hour, cooling on ice, the incubation procedure was resumed. Bupropion, a CYP2B6 substrate [19–21], and benzydamine, an FMO substrate [34], were used as positive and negative control substrates to assess CYP, FMO and CYP2B6 inhibition. Samples not preincubated with chemical inhibitors nor heated to 45 ℃ were used as 100% TATP-OH formation. Inhibition studies were quenched after 15 min incubation.

Recombinant CYP and FMO enzymes were employed to identify the isoform responsible for the NADPH-dependent metabolism. Human bactosomes expressed in *E. coli* were used for CYP isoform identification; CYP1A2, CYP2B6, CYP2C9, CYP2C19, CYP2D6, CYP2E1 and CYP3A4 (100 pmol CYP/mL) were tested. Human supersomes expressed in insect cells were used for FMO isoform identification; FMO1, FMO3 and FMO5 (100 µg protein/mL) were examined. Both TATP and TATP-OH were tested as the substrate (10 µg/mL) in the incubation mixture with the recombinant enzymes. Negative control incubations were done in *E. coli* control or insect cell control. Positive control incubations were done in HLM (200 pmol CYP/mL), which contains all CYP and FMO enzymes. Recombinant enzymes studies were quenched after 10 min incubation.

Recombinant UGT enzymes were used to identify the isoform responsible for phase II metabolism. Human supersomes expressed in insect cells were used for UGT isoform identification; UGT1A1, UGT1A3, UGT1A4, UGT1A6, UGT1A9 and UGT2B7 were examined. The incubation mixture was similar to the glucuronidation incubation previously described, except that saccharolactone was not added [35]; 500 µg protein/mL was used; and the substrate was 10 µg/mL TATP-OH. Negative control incubations were done in insect cell control. Positive control incubations were done in HLM (1 mg protein/mL), which contained all UGT enzymes. Glucuronidation with recombinant enzymes was quenched after 2 h incubation.

### Enzyme kinetics

Kinetics experiments were done to determine the affinity of the enzyme CYP2B6 to the substrate TATP. The human CYP2B6 bactosomes used contained human CYP2B6 and human CYP reductase coexpressed in *E. coli*, supplemented with purified human cytochrome b_5_. Cytochrome P450 reductase is responsible for the transfer of electrons from NADPH to CYP, a task sometimes extended to cytochrome b_5_ [11]. The incubation mixture (1 mL) contained 10 mM phosphate buffer (pH 7.4), 2 mM MgCl_2_, 50 nM rCYP2B6, 1 mM NADPH, and various concentrations of 0.1 to 20 µM TATP. The reaction was initiated by adding TATP after a 3 min pre-equilibration and stopped at different end points (up to 5 min) to determine rate of TATP hydroxylation. The rate of TATP hydroxylation in lungs was also investigated by incubating TATP (100 µM) in the incubation mixture containing 1 mg/mL HLM or HLungM, instead of rCYP2B6, for up to 10 min.

TATP-OH metabolism by CYP2B6 was evaluated by incubating 10 µg/mL TATP-OH in CYP2B6 bactosomes according to the above procedure, with or without NADPH, except that the buffer was preoxygenated so that the incubation could be performed in a closed vessel. Aliquots were removed and quenched at different time points, up to 30 min. TATP-OH depletion by HLM was determined using the same procedure, except that 1 µM TATP-OH and up to 60 min reaction times were used.

### Urine analysis

Laboratory personnel testing TATP are constantly exposed to this volatile compound. Explosive sensitivity experiments, such as drop weight impact tests, are done in a small brick-walled room for explosivity precautions, unlike synthesis reactions which are performed inside a fume hood for coverage protection. Also, portable explosive trace detection devices that are meant to be used in the field are tested as such, which also contribute to exposure. Urine from laboratory workers was tested for TATP and its metabolites after TATP exposure in the laboratory environment. Urine was collected at the beginning of the work week and 2 h after performing activities that could lead to high TATP exposure. To determine the longevity of TATP in the body, urine from the day following TATP exposure was also tested. The fresh urine was cleaned and concentrated for analysis using solid-phase extraction. Restek RDX column (Restek, Bellefonte, PA, USA) was conditioned with 6 mL of methanol, followed by 6 mL of water, and sample introduction (20–250 mL urine). The sample was washed with two cycles of 3 mL methanol/water (50:50, v/v). Extraction was achieved with two cycles of 1 mL acetonitrile. Both eluents were tested by LC–MS, because the lipophilic TATP and TATP-OH were extracted with acetonitrile, but the hydrophilic TATP-*O*-glucuronide was present in the methanol/water wash.

## Results

### Metabolite identification

**Fig. 1:**
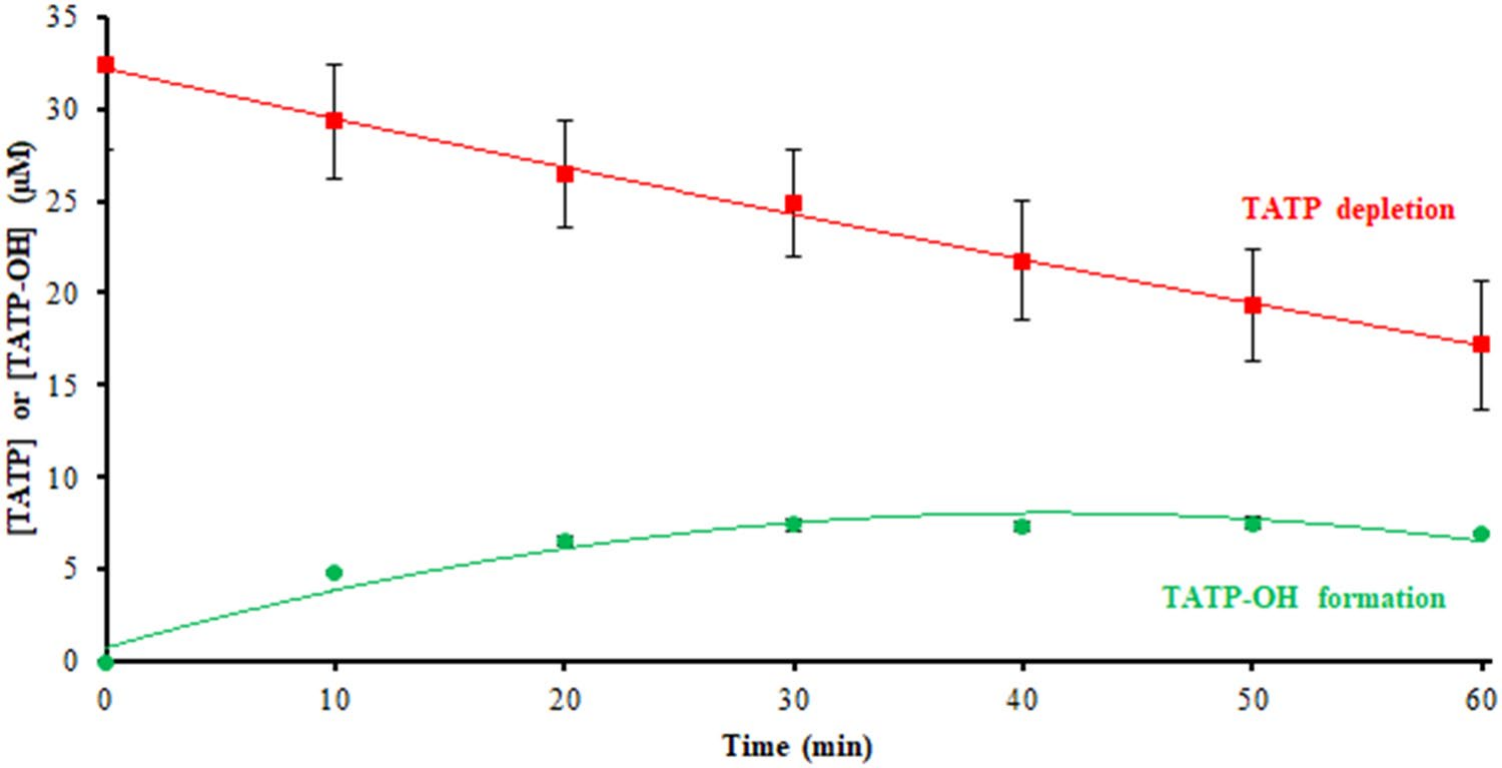
Triacetone triperoxide (TATP) biotransformation into hydroxy-TATP (TATP-OH) monitored over time in human liver microsomes (HLM), performed in triplicate

**Fig. 2:**
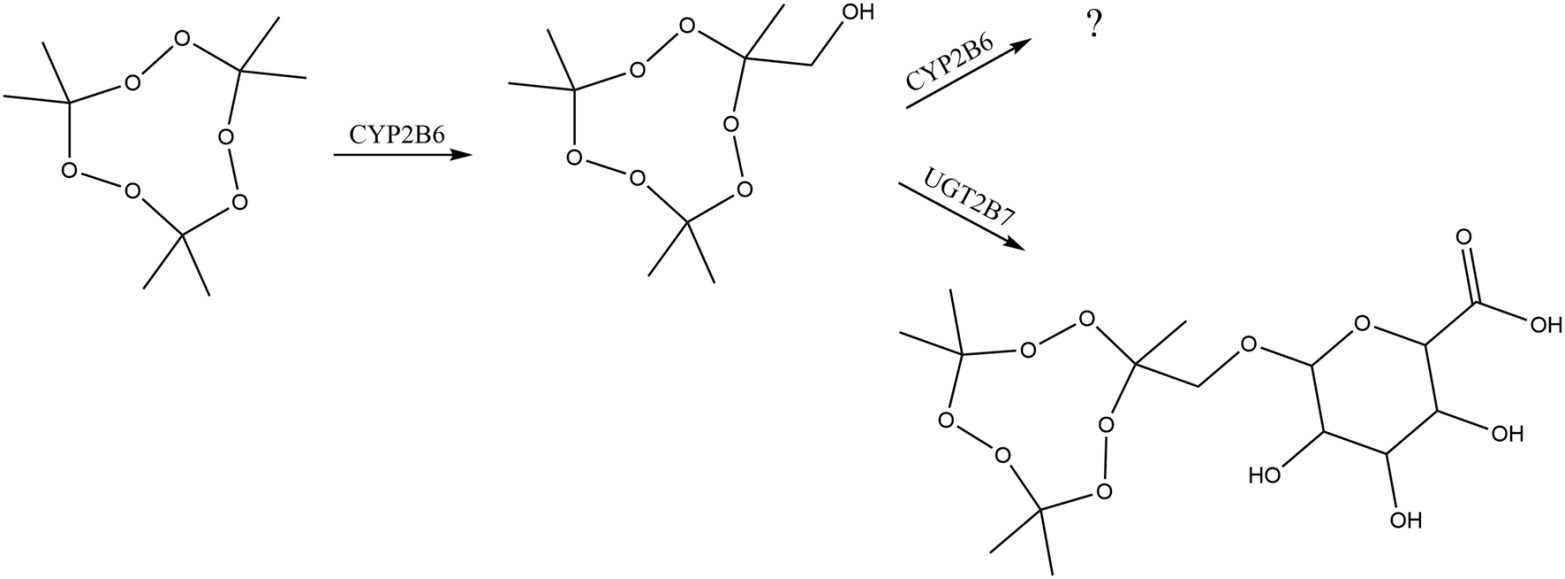
TATP metabolic pathways in HLM. *CYP* cytochrome P450, *UGT* uridine diphosphoglucuronosyltransferase

When TATP was incubated in HLM, TATP was depleted, and one observable product, TATP-OH, was formed over time (Fig. 1). Metabolism of TATP in HLM consists of hydroxylation at one methyl group with the peroxide bonds and nine-membered ring structure preserved (Fig. 2) [15]. Opsenica and Solaja [12] reported various monohydroxylated and dihydroxylated products during microsomal incubations with cyclohexylidene and steroidal mixed tetraoxanes where the peroxide bond was also preserved. A TATP-OH standard (Fig. S4) was chemically synthesized to confirm the metabolite by retention time and mass-to-charge ratio (*m/z*). TATP-OH, identified as [TATP-OH + NH_4_]^+^ (*m/z* 256.1391) by accurate mass spectrometry, increased in incubation samples as time progressed. Attempts to confirm the hydroxylated metabolite with the deuterated substrate were unsuccessful due to the persistence of a contaminant with the same mass that the metabolite would have had, even in samples where TATP-*d*_18_ was not used as the substrate. As previously observed, TATP incubations in different species (dogs and rats) yielded the same metabolite, TATP-OH (Fig. S5) [15]. Other suspected metabolites, including the dihydroxy-species and additional oxidation of the TATP-OH to the aldehyde and carboxylic acid were not observed. Small polar molecules, such as acetone and hydrogen peroxide, the synthetic reagents of TATP, could not be chromatographically separated or are below the lower mass filter limit.

**Fig. 3:**
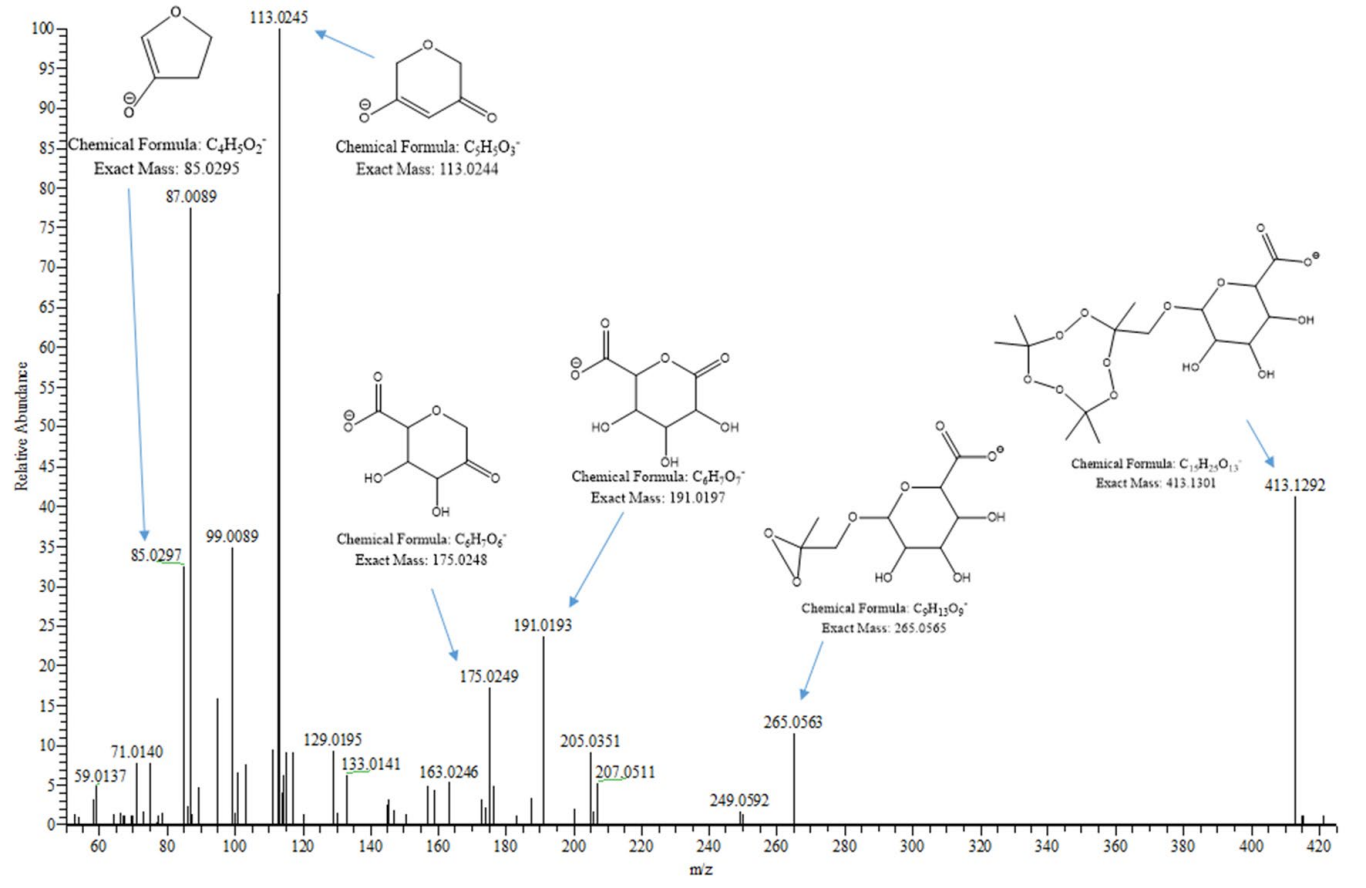
Product ion spectrum of [TATP-*O*-glucuronide − H]^−^ (*m/z* 413.1301), fragmented with 35 eV using electrospray ionization in negative mode (ESI–). Proposed structures are shown

TATP was investigated for phase II metabolism routes of glutathione and glucuronide conjugation. Incubation in HLM with GSH produced no detectable glutathione metabolite conjugates, indicating that TATP is most likely not a substrate for microsomal GSTs (Fig. S6). When TATP was incubated with UDPGA, the TATP-OH glucuronic acid metabolite (TATP-*O*-glucuronide) was observed (Fig. 2). The *m/z* 432.1712 for [TATP-*O*-glucuronide + NH_4_]^+^ was observed at very low levels after 2 and 3 h of incubation. When the sample was dried and reconstituted in low volume, the intensity of [TATP-*O*-glucuronide + NH_4_]^+^ increased, but TATP and TATP-OH were evaporated along with the solvent. TATP and TATP-OH are volatile, limiting their use in quantification experiments since sample concentration is not feasible [5]. However, TATP-*O*-glucuronide is a non-volatile TATP derivative that is amenable to sample preparation. Formation of TATP-*O*-glucuronide over time was monitored, using concentrated samples, as an intensity increase of *m/z* 432.1712 (Fig. S7). Even though TATP and TATP-OH generally form ammonia adducts under positive ion APCI, the glucuronide favors negative ion mode ESI. The *m/z* 413.1301 for [TATP-*O*-glucuronide − H]^−^ was easily seen at 1–3 h without sample concentration. The common fragments of glucuronic acid, *m/z* 175, 113, and 85, were observed in the fragmentation pattern of *m/z* 413.1301, confirming the presence of a glucuronic acid conjugate (Fig. 3) [36]. TATP-*O*-glucuronide was not observed in negative controls without UDPGA or NADPH, indicating that TATP-OH must be formed and then be further metabolized into TATP-*O*-glucuronide. Glucuronide conjugates are highly polar compounds that are easily eliminated by the kidney, suggesting that TATP-*O*-glucuronide would likely progress via urinary excretion [11].

### Enzyme identification

TATP was only metabolized into TATP-OH in HLM in the presence of NADPH, indicating that the metabolism is NADPH-dependent. The predominant microsomal enzymes that require NADPH for activity are CYP and FMO [11].

**Fig. 4:**
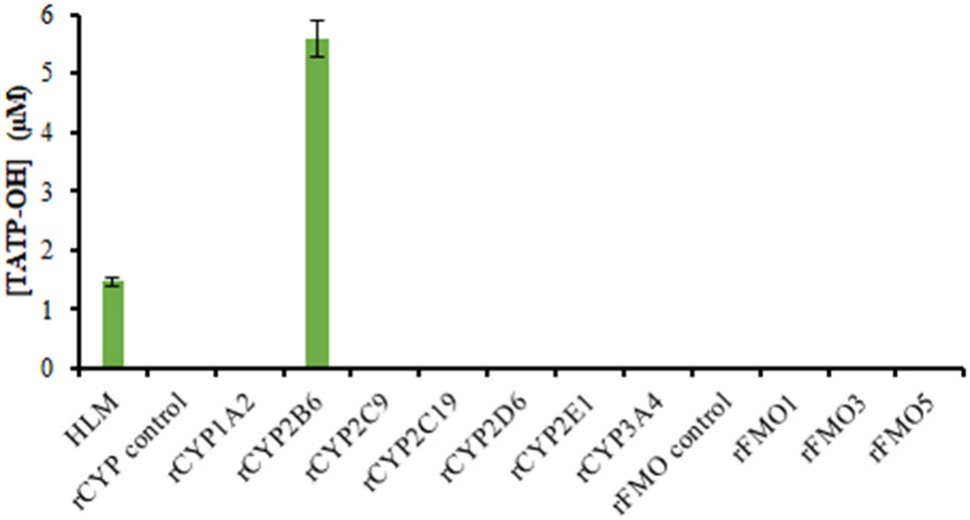
TATP-OH formation from TATP incubations with recombinant cytochrome P450 (rCYP) and recombinant flavin monooxygenase (rFMO). Experiments with rCYP or rFMO consisted of 10 µg/mL TATP incubated with 10 mM phosphate buffer (pH 7.4), 2 mM MgCl_2_ and 1 mM NADPH. Incubations were done in triplicate and quenched at 10 min

The usual roles of CYP are hydroxylation of an aliphatic or aromatic carbons, epoxidation of double bonds, heteroatom oxygenation or dealkylation, oxidative group transfer, cleavage of esters and dehydrogenation reactions [11]. 1-Aminobenzotriazole is considered a general mechanism-based inhibitor of CYPs, initiated by metabolism into benzyne, which irreversibly reacts with the CYP heme [28, 29]. When CYP activity was inhibited by 1-aminobenzotriazole, TATP-OH formation was also inhibited, with only 3.6% formed (Table 2), suggesting that CYP is involved in the hydroxylation of TATP. To support this evidence and to narrow down the CYP isoform catalyzing TATP hydroxylation, TATP was incubated with rCYPs (Fig. 4, Table S1). The CYPs selected for testing are responsible for the metabolism of 89% of common xenobiotics [37]. TATP-OH was not observed when TATP was incubated with CYP1A2, CYP2C9, CYP2C19, CYP2D6, CYP2E1 and CYP3A4. TATP hydroxylation was performed exclusively by CYP2B6, with 5.6 ± 0.3 µM TATP-OH produced in 10 min. CYP2B6 has been found to metabolize endoperoxides by hydroxylation, as observed for TATP [13, 14]. HLM, which contains CYP2B6, also exhibited TATP hydroxylation (1.5 ± 0.1 µM).

**Table 2:** Average % metabolites formed (triplicates) in 15 min incubations with chemical inhibitors or heat

Metabolite formation in 15 min	Percent found						
	Inhibitor preincubated in HLM for 30 min					HLM preheated for 5 min	
	No inhibitor	1-ABT	MMI	TIC	TATP or BUP	37 ℃	45 ℃
TATP-OH (%)	100 ± 5	3.6 ± 0.7	34 ± 2	4.8 ± 0.7	125 ± 21	100 ± 10	69 ± 4
BUP-OH (%)	100 ± 3	23 ± 1	62 ± 2	9.6 ± 0.4	62 ± 2	100 ± 6	99 ± 2
BZD-NO (%)	100 ± 2	97 ± 1	48 ± 1	98 ± 3	N/A	100 ± 2	44 ± 2

*1-ABT* 1-aminobenzotriazole (CYP inhibitor), *CYP* cytochrome P450, *FMO* flavin monooxygenase, *HLM* human liver microsomes, *MMI* methimazole (FMO inhibitor), *TIC* ticlopidine (CYP2B6 inhibitor) (for other abbreviations, see Table 1)

Since DLM studies indicated that TATP is metabolized by CYP2B11 [15], it was not surprising that another CYP2B subfamily enzyme, CYP2B6, metabolizes TATP in humans. CYP2B6 metabolism of TATP was further investigated by incubating TATP with ticlopidine, a mechanism-based inhibitor of CYP2B6 [32, 33]. When CYP2B6 activity was inhibited by ticlopidine, TATP-OH formation was also inhibited by 95% (Table 2), further supporting the primary involvement of CYP2B6 in the metabolism of TATP. Bupropion hydroxylation is catalyzed by CYP2B6; therefore, it was chosen as a positive control for CYP and CYP2B6 inhibition tests [28]. Hydroxybupropion formation was inhibited by 77 and 90% when incubated with 1-aminobenzotriazole and ticlopidine, respectively (Table 2).

FMO catalyzes oxygenation of nucleophilic heteroatoms, such as nitrogen, sulfur, phosphorous and selenium [34, 38]. Although FMO involvement in the hydroxylation of the TATP methyl group was unlikely, it seemed prudent to examine this enzyme class. Addition of methimazole, an FMO competitive inhibitor [30, 31], caused a decrease in TATP-OH formation by 66% (Table 2), but this is not necessarily direct inhibition of TATP metabolism by FMO since methimazole has been reported to reduce CYP2B6 activity by up to 80% [34, 39]. While this result was inconclusive about the FMO contributions to TATP metabolism, it could be considered further support for the role of CYP2B6. TATP was incubated with available rFMOs: FMO1, FMO3 and FMO5 (Fig. 4, Table S1). FMO1 is expressed in adults in the kidneys, and it should not contribute to the liver metabolism of TATP [34]; indeed, none appeared to be involved as no TATP-OH was produced (positive control, Fig. S8). Since FMO is inactivated by heat [11, 34], TATP was incubated in HLM pre-heated to 45 ℃ for 5 min. Table 2 compares the formation of TATP-OH at 37 ℃ to that at 45 ℃ and to the *N*-oxidation of the positive control, benzydamine. Although, there was a decrease in TATP hydroxylation, the inhibitory effect was not as significant as compared to the decrease in benzydamine *N*-oxidation, which is catalyzed by FMO.

TATP-OH appears to be metabolized by HLM in an NADPH-dependent manner; therefore, TATP-OH was incubated for 10 min with recombinant enzymes (CYP and FMO) to determine which isoform is responsible for this secondary phase I metabolism (Table 3). Although TATP-OH is not nearly as volatile as TATP, some TATP-OH was lost in all incubations (i.e., 37 ℃); however, notable depletion (by 40%) was observed only with CYP2B6. TATP-OH depletion in CYP2B6 was faster in the presence of NADPH than in its absence, supporting metabolism by CYP2B6 (Fig. S9). Unfortunately, no subsequent metabolite was identified.

**Table 3:** Average % TATP-OH remaining (triplicates) after 10 min incubation in recombinant enzymes

Incubation matrix	Percent TATP-OH remaining
HLM	69 ± 5
rCYP control	76 ± 4
rCYP1A2	77 ± 2
rCYP2B6	60 ± 3
rCYP2C9	71 ± 8
rCYP2C19	76 ± 5
rCYP2D6	76 ± 5
rCYP2E1	73 ± 5
rCYP3A4	78 ± 6
rFMO control	69 ± 3
rFMO1	78 ± 12
rFMO3	78 ± 6
rFMO5	72 ± 7

*rCYP* recombinant cytochrome P450, *rFMO* recombinant flavin monooxygenase

**Fig. 5:**
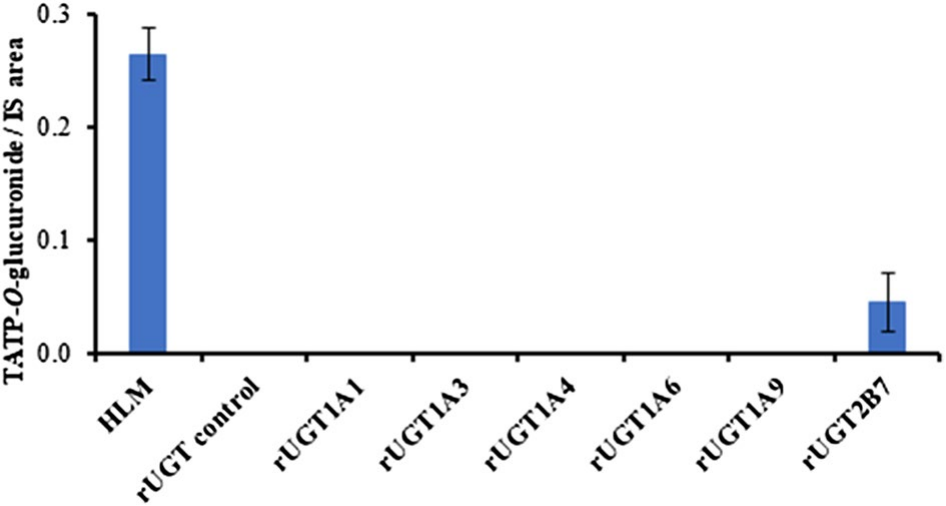
TATP-*O*-glucuronide formation from TATP-OH incubations with recombinant uridine diphosphoglucuronosyltransferase (rUGT). Experiments with rUGT consisted of 10 µg/mL TATP-OH incubated with 10 mM phosphate buffer (pH 7.4), 2 mM MgCl_2_, 50 µg/mL alamethicin, 1 mM NADPH, and 5.5 mM uridine diphosphoglucuronic acid. Glucuronidation done in triplicate and quenched at 2 h. Quantification was done using area ratio TATP-*O*-glucuronide/internal standard 2,4-dichlorophenoxyacetic acid

To identify which isoform is responsible for TATP glucuronidation, TATP-OH was incubated with the most clinically relevant rUGTs (Fig. 5, Table S2) [40]. Glucuronidation was not observed with UGT1A1, UGT1A3, UGT1A4, UGT1A6 and UGT1A9. TATP-*O*-glucuronide was formed only in UGT2B7 incubations with 0.05 ± 0.03 area count relative to IS produced after 2 h of incubation. HLM, which contains UGT2B7, also displayed TATP-*O*-glucuronide (0.26 ± 0.02 area count relative to IS). Relative quantification of TATP-*O*-glucuronide was done by area ratio to the IS because a TATP-*O*-glucuronide standard is not available. Endoperoxide glucuronidation by UGT2B7 has been reported, in which urine analysis of patients treated with artesunate, an artemisinin derivative, found dihydroartemisinin-glucuronide to be the principal metabolite excreted [16].

### Enzyme kinetics

**Fig. 6:**
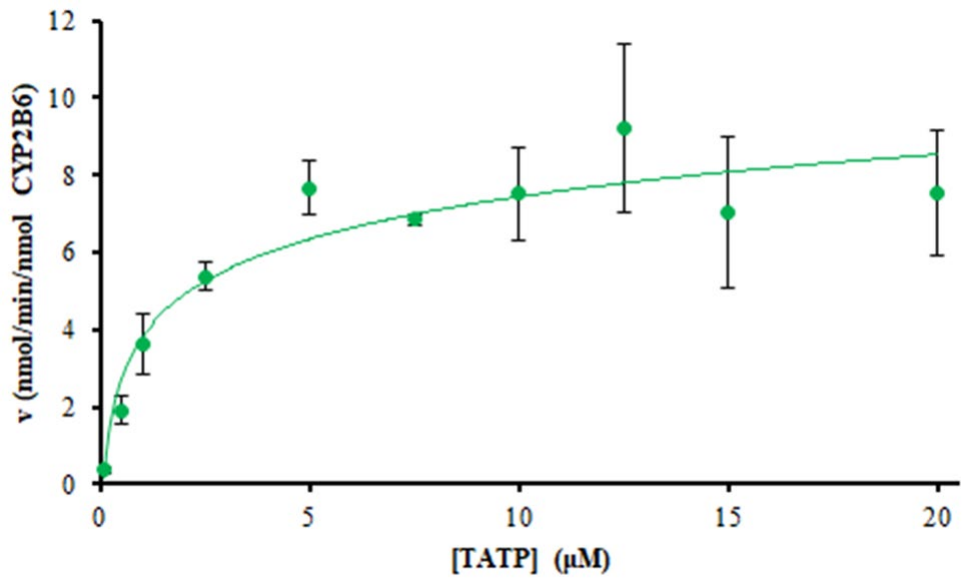
Rate of TATP hydroxylation by CYP2B6 versus TATP concentration. Incubations of various TATP concentrations consisted of 50 pmol rCYP2B6/mL with 10 mM phosphate buffer (pH 7.4), 2 mM MgCl_2_ and 1 mM NADPH. Incubations were done in triplicate and quenched every min up to 5 min

Rate of TATP hydroxylation by CYP2B6 was evaluated by plotting concentration of TATP-OH formed over time. The initial rate of TATP hydroxylation at various TATP concentrations was used to estimate enzyme kinetics using the Michaelis–Menten model: $v\, = \,V_{{\max}} \, \times \,\left[ S \right]/\left( {K_{{\text{m}}} \, + \,\left[ S \right]} \right)$. Here, [*S*] is the substrate (TATP) concentration, *V*_max_ is the maximum formation rate, *K*_m_ is the substrate concentration at half of *V*_max_, and *k*_cat_ is the turnover rate of an enzyme–substrate complex to product and enzyme [41]. The kinetic constants were obtained using nonlinear regression analysis on GraphPad Prism software (version 8.2.1). The Michaelis–Menten evaluation for TATP hydroxylation by CYP2B6 (Fig. 6) yielded *K*_m_ of 1.4 µM; *V*_max_ of 8.7 nmol/min/nmol CYP2B6; and *k*_cat_ of 174 min^− 1^. Linearized models, such as Lineweaver–Burk (Fig. S10), Eadie–Hofstee (Fig. S11) and Hanes–Woolf (Fig. S12), give similar values.

The low *K*_m_ indicates TATP has a high affinity for CYP2B6 [42]. Table 2 shows that TATP inhibits bupropion hydroxylation by CYP2B6, with only 62% hydroxybupropion formation in 15 min. However, in the presence of bupropion, TATP-OH formation was enhanced, with 125% formed compared to the reaction uninhibited by bupropion (Table 2). CYP2B6 preference for TATP affects the metabolism of bupropion, but further testing is needed to establish the specific type of inhibition.

Using the Michaelis–Menten parameters, in vitro intrinsic clearance ($Cl_{{{\text{int}}}} \, = \,V_{{\max}} /K_{{\text{m}}}$) was calculated to be 6.13 mL/min/nmol CYP2B6 [11, 43, 44]. Scale-up of the *Cl*_int_ to yield intrinsic clearance on a per kilogram body weight was done using values of 0.088 nmol CYP2B6/mg microsomal protein, 45 mg microsomal protein/g liver wet weight and 20 g liver wet weight/kg human body weight [43]. Taking that into account, the scale-up *Cl*_int_ was calculated to be 485 mL/min/kg.

In vivo intrinsic clearance (*Cl*) is the ability of the liver to metabolize and remove a xenobiotic, assuming normal hepatic blood flow (*Q* = 21 mL/min/kg [43, 45]) and no protein binding [43]. *Cl* can be extrapolated using the well-stirred model excluding all protein binding as $Cl\, = \,Q\, \times \,Cl_{{\text{int}}} /Q\, + \,Cl_{{\text{int}}}$ [43]. The in vivo intrinsic clearance of TATP was estimated as 20 mL/min/kg. Compared to common drugs, TATP has a moderate clearance [46].

**Fig. 7:**
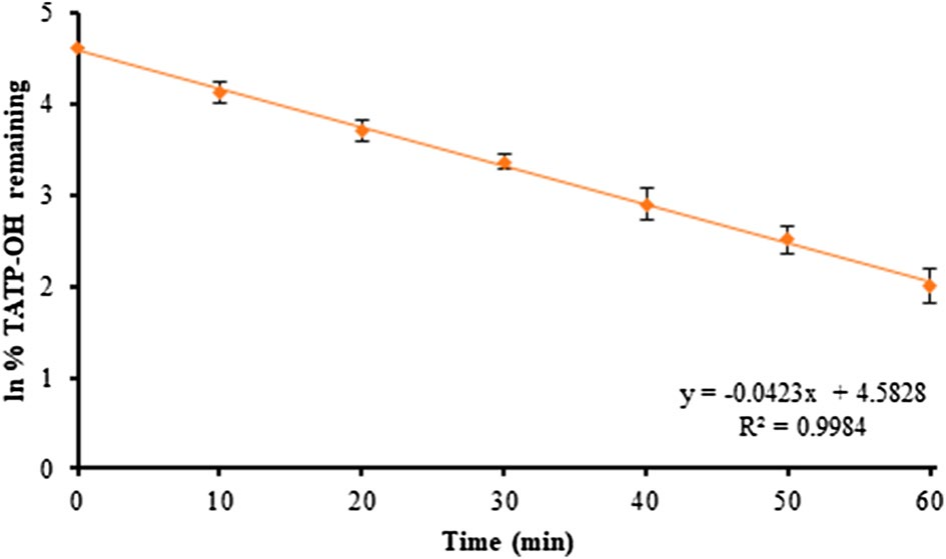
Natural log of TATP-OH percent remaining in HLM versus time. TATP-OH (1 µM) incubated in 1 mg/mL HLM with preoxygenated 10 mM phosphate buffer (pH 7.4), 2 mM MgCl_2_ and 1 mM NADPH. Incubations were done in closed vials, in triplicate and quenched every 10 min up to 1 h

TATP-OH kinetics were also investigated by substrate depletion. Substrate depletion was plotted as the natural log of substrate percent remaining over time (Fig. 7). Half-life (*t*_1/2_) was calculated to be 16 min, as the natural log 2 divided by the negative slope of the substrate depletion plot. In vitro intrinsic clearance (*Cl*_int_) can also be estimated using half-life as *Cl*_int_ = (0.693/*t*_1/2_) × (incubation volume/mg microsomal protein) [47, 48]. The in vitro intrinsic clearance of TATP-OH was estimated as 0.042 mL/min/mg. Even though we identified two metabolic pathways for TATP-OH, it appears to be cleared slower than TATP.

### Lung metabolism

Inhalation is the most probable pathway for systemic exposure since TATP is both volatile and lipophilic. With passive diffusion into the bloodstream being very possible, TATP metabolism in the lung was also investigated. TATP was incubated in lung and liver microsomes for comparison of metabolic rate. The results, shown in Table 4, indicated that TATP hydroxylation in the lungs was negligible. Though CYP2B6 gene and protein are expressed in the lungs, enzyme activity in lungs is minimal as compared to the liver, limiting TATP metabolism [49, 50]. This suggests that TATP is most likely distributed through the blood to the liver for metabolism. News reported that traces of TATP was found in the blood samples extracted from the 2016 Brussels suicide bombers [51]. This indicates the possibility of using blood tests as forensic evidence for TATP exposure.

**Table 4:** Rate of TATP hydroxylation (triplicates) in human liver microsomes versus human lung microsomes

Human microsomes	Rate of TATP-OH formation (nmol/min/mg)
Liver	0.425 ± 0.06
Lung lot1710142	< LOQ
Lung lot1410246	< LOQ

*< LOQ* lower than the limit of quantification

### In vivo human urine analysis

**Fig. 8:**
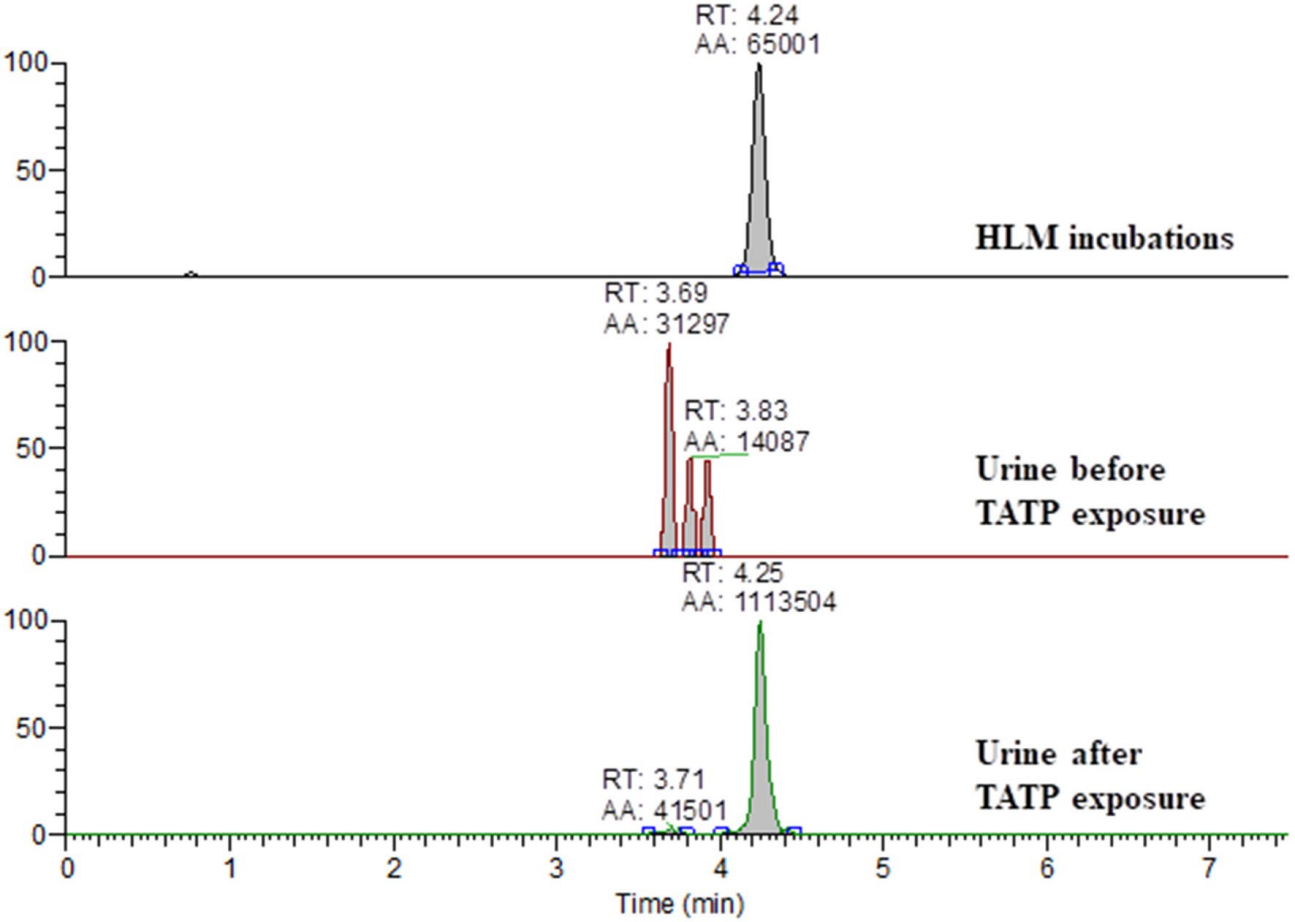
Extracted ion chromatogram of [TATP-*O*-glucuronide − H]^−^ (*m/z* 413.1301) in HLM 2 h after incubation with TATP, and in human urine, before TATP exposure and 2 h after TATP exposure

Laboratory workers, who normally work with TATP on a daily basis, performing tasks like explosive sensitivity testing, were screened for TATP exposure. These laboratory workers volunteered to collect their urine before and after exposure to TATP vapor. Since the health effects of TATP exposure are unknown, to minimize any additional risks to these workers, this pilot study was performed in duplicates using only three volunteers to establish some reproducibility. TATP and TATP-OH were not observed in the urine of any of the workers. However, TATP-*O*-glucuronide was present in all urine samples collected 2 h after TATP exposure (Fig. 8). Two out of the three volunteers still showed TATP-*O*-glucuronide in the urine collected the next day (Table 5). TATP-*O*-glucuronide was identified in human urine samples as both [TATP-*O*-glucuronide − H]^−^ and [TATP-*O*-glucuronide + NH_4_]^+^. The presence of TATP-*O*-glucuronide in the urine of all three volunteers is summarized in Table 5.

**Table 5:** Summary of TATP-*O*-glucuronide presence in human urine (duplicates) in vivo

Human	*m/z* 413.1301			*m/z* 432.1712		
	#1	#2	#3	#1	#2	#3
Before TATP exposure	−	−	−	−	−	−
Two hours after TATP exposure	+	+	+	+	+	+
One day after TATP exposure	+	+	−	+	−	−

TATP glucuronide is observed as [TATP-*O*-glucuronide − H]^−^ (*m/z* 413.1301) and [TATP-*O*-glucuronide + NH_4_]^+^ (*m/z* 432.1712, less sensitive). Only one trial performed on next day samples

## Discussion

As described before, TATP is the explosive of choice by terrorists because it is easily synthesized from household items [1, 2]. In our previous study, we have clarified that TATP-OH is produced as an in vitro metabolite from TATP in dogs [15], because canines are currently one of the most reliable detection techniques used to find an explosive [52]. In the present article, the study has been conducted in continuation of our previous findings on both in vitro and in vivo metabolism of TATP in humans (Fig. 2).

Because TATP has high volatility, it is likely to be absorbed into the body by inhalation; however, no appreciable metabolism in the lung was observed in either dog [15] or human microsomes (Table 4). Therefore, systemic exposure and subsequent liver metabolic clearance were presumed. Across three species, dog, rat, and human, TATP was metabolized in liver microsomes by CYP to TATP-OH (Fig. S5). Using recombinant enzymes, we have previously established that CYP2B11 is responsible for this metabolism in dogs [15]. Interestingly, human CYP2B6 appears to be the major phase I enzyme responsible for the same metabolism (Fig. 4). TATP hydroxylation by CYP2B6 kinetics determined *K*_m_ and *V*_max_ as 1.4 µM and 8.7 nmol/min/nmol CYP2B6, respectively (Fig. 6)_._ Though heat inactivation and chemical inhibition of FMO appeared to affect TATP hydroxylation (Table 2), incubations with recombinant FMO suggest that FMO was not forming TATP-OH (Fig. 4). Methimazole, an FMO inhibitor, inhibited TATP-OH formation (Table 2); but, inhibition of bupropion by this chemical inhibitor suggests that methimazole also inhibits CYP2B6 activity [39].

When incubated together, TATP and bupropion appear to compete for CYP2B6 metabolism, with bupropion hydroxylation being inhibited by 38% in the presence of TATP (Table 2). Considering CYP2B6 expression in the liver is low and exhibits broad genetic polymorphisms, CYP2B6 activity can be widely affected if TATP affects the metabolism of other compounds, like bupropion [53, 54]. TATP may be a serious perpetrator for drug-drug interactions for compounds cleared by CYP2B6 [55, 56].

In vitro clearance of TATP was calculated as 0.54 mL/min/mg protein with hepatic in vivo extrapolation to 20 mL/min/kg. In vitro clearance of TATP-OH was estimated, using substrate depletion, as 0.038 mL/min/mg protein (Fig. 7). We also established the clearance of TATP in dogs as 0.36 mL/min/mg protein in our previous study in canine microsomes [15], which is significantly relevant to K9 units, where the dog and human handler are both exposed to the explosive.

Investigation into the next step on the metabolic pathway suggested that TATP-OH is further metabolized by CYP2B6 (Table 3), but a secondary phase I metabolite was not identified. No glutathione adducts of any TATP metabolism products were observed in the microsomal incubations with GSH (Fig. S6). However, glucuronidation converted TATP-OH to TATP-*O*-glucuronide in HLM with UGT2B7 specifically catalyzing this reaction (Fig. 5). Considering glucuronides are often observed as urinary metabolites, the presence of TATP-*O*-glucuronide in urine can be exploited as an absolute marker of exposure to TATP, which can be used as forensic evidence of TATP illegal use.

Urine from scientists working to prevent terrorist attacks by synthesizing, characterizing and detecting TATP, who are inevitably exposed to this volatile compound were negatively tested for TATP and TATP-OH, but TATP-*O*-glucuronide was present at high levels in their fresh urine (Fig. 8). In one out of the three volunteers, TATP-*O*-glucuronide was not observed in the urine collected the day after TATP exposure (Table 5), suggesting TATP to TATP-*O*-glucuronide in vivo clearance occurs within about a day depending on the exposure level. TATP-*O*-glucuronide presence in the urine of all three volunteers shows good in vivo correlation to in vitro data.

Like TATP (hydrophilicity expressed as TPSA = 55.38 and lipophilicity expressed as cLogP = 3.01, calculated using PerkinElmer ChemDraw Professional version 16.0.1.4), TATP-OH is lipophilic with TPSA and cLogP of 75.61 and 1.72, respectively. TATP-*O*-glucuronide, on the other hand, is hydrophilic with TPSA and cLogP of 171.83 and 0.32, respectively. The increase in TPSA and decrease in cLogP from TATP to TATP-*O*-glucuronide accounts for the glucuronide greater water solubility and facilitated excretion [11], thus explaining the presence of only TATP-*O*-glucuronide in urine.

Even though working with TATP falls under the protection of several standard operating procedures to handling explosives, considering the breathing exposure that these laboratory workers revealed, implementation of precautionary measures to absorption by inhalation, such as the use of respirators, should be considered. Such detection of TATP-*O*-glucuronide is also useful for judicial authorities to raise a scientific evidence for exposure to TATP of terrorists and/or related individuals.

This paper is the first to examine some aspects of TATP human ADMET, elucidating the exposure, metabolism and excretion of TATP in humans. However, the detailed pharmacological and toxicological studies remain to be explored.

## Conclusions

This article dealt with in vitro and in vivo studies of TATP metabolism in humans. TATP is highly volatile and easily introduced into human body via aspiration. By this study, TATP was found to be metabolized into TATP-OH by the action of CYP2B6, followed by glucuronidation of TATP-OH catalyzed by UGT2B7; the resulting TATP-*O*-glucuronide was found to be excreted into urine in live humans. After extracting the TATP conjugate from urine specimens, it can be analyzed by HPLC–MS/MS, which gives scientific evidence for exposure to TATP. This evidence can be useful to prove exposure of persons, such as terrorists, to TATP for judicial authorities. Although this study includes the metabolism of TATP and also an analytical method to detect the TATP-*O*-glucuronide, the toxicology of TATP remains to be explored.

### Electronic supplementary material

Below is the link to the electronic supplementary material.

Supplementary file1 (PDF 530 kb)
