# Supplementary material for: In vitro and in vivo studies of triacetone triperoxide (TATP) metabolism in humans
Source: Forensic Toxicol. 2020 Jul 14;39(1):59–72. doi: 10.1007/s11419-020-00540-z (PMC10615923; doi:10.1007/s11419-020-00540-z)
Supplement: Supplementary file 1 — Supplementary file1 (PDF 530 kb) [file 11419_2020_540_MOESM1_ESM.pdf]

In vitro and in vivo studies of triacetone triperoxide (TATP) metabolism in humans

Supplementary Material

Michelle Gonsalves[1]; Kevin Colizza[1]; James L. Smith[1]; Jimmie C. Oxley[1*]

[1] Department of Chemistry, University of Rhode Island, 140 Flagg Rd, Kingston, RI 02881, USA.
[*] Corresponding author:
Address: 140 Flagg Rd, Kingston, RI 02881, USA.
Phone number: 401- 874-2103
Email: joxley@chm.uri.edu

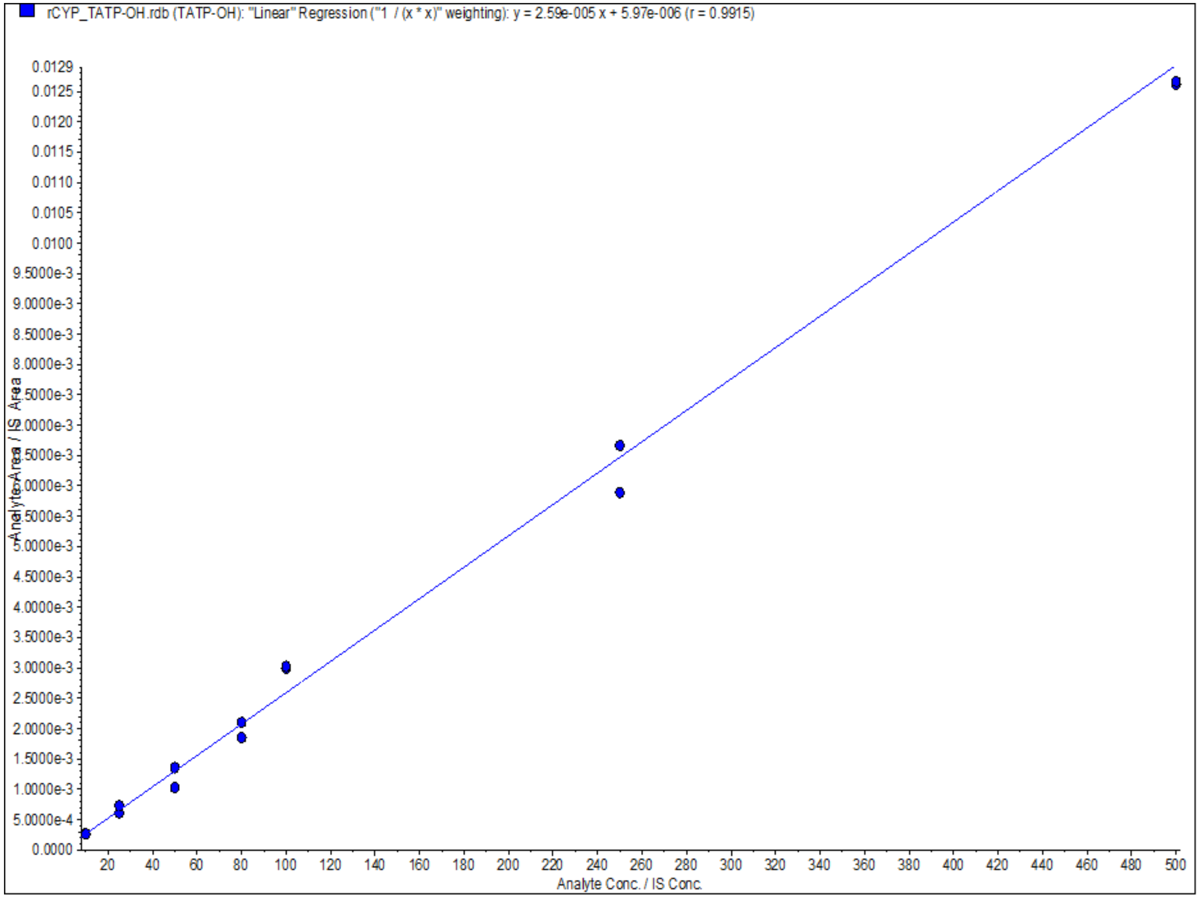

Fig. S1 Hydroxy-triacetone triperoxide (TATP-OH) standard curve from 10 to 500 ng/mL

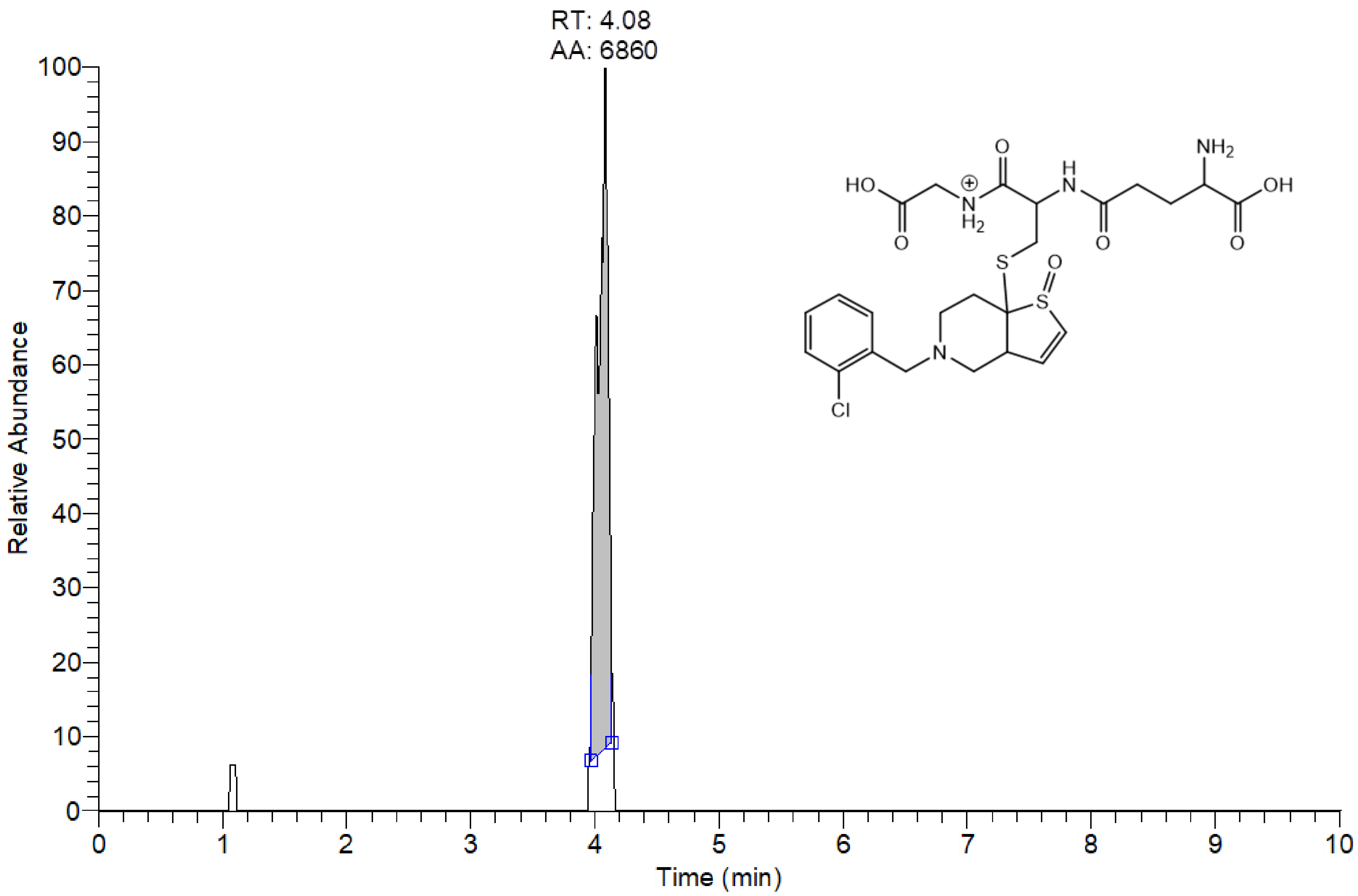

Fig. S2 Extracted ion chromatogram of ticlopidine glutathione metabolite from ticlopidine metabolized by gluthathione S-trasferase (GST) after 1h incubation, presented as a GST positive control

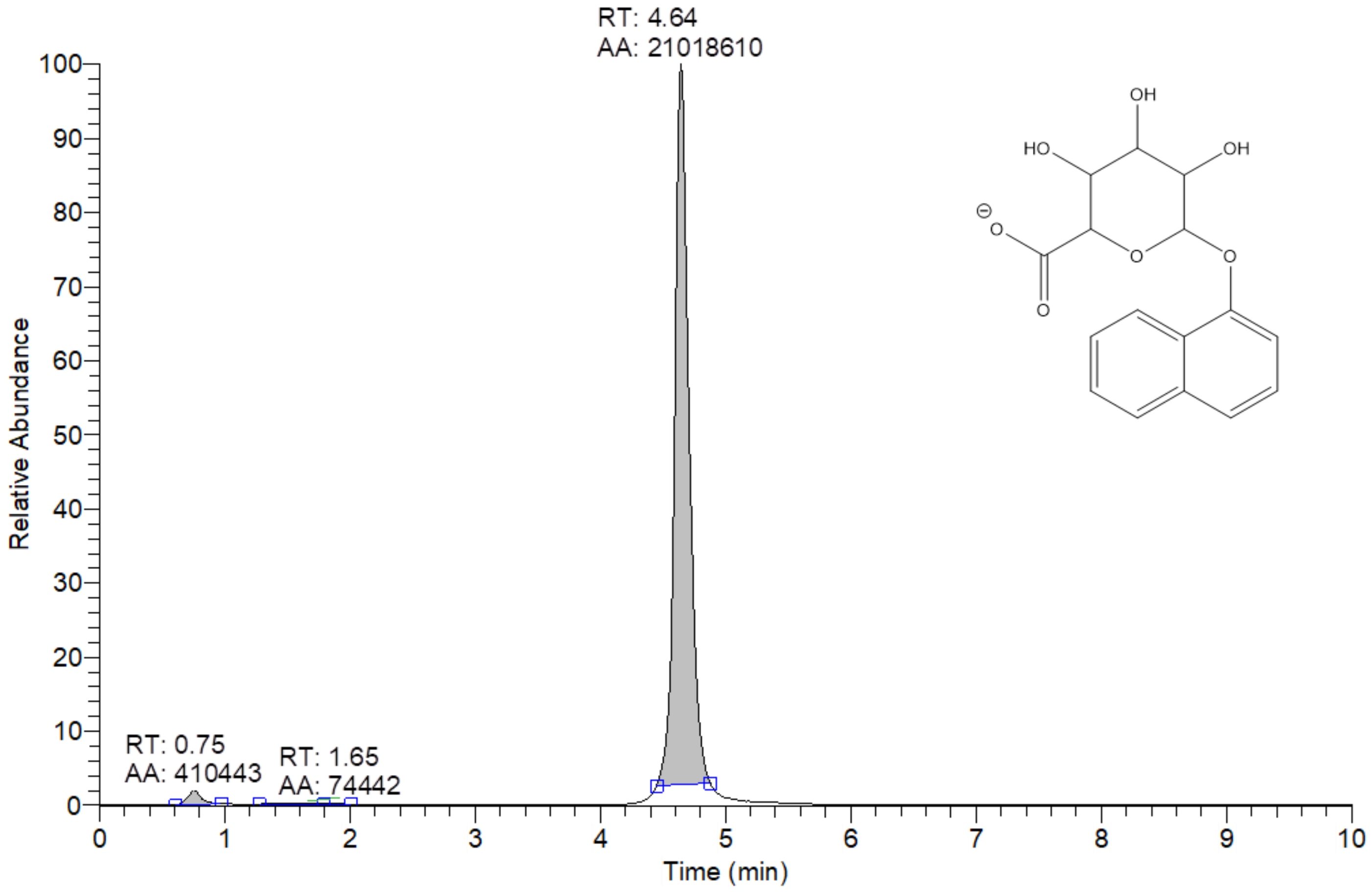

Fig. S3 Extracted ion chromatogram of naphthol glucuronide metabolite from 1-naphthol metabolized by uridine diphosphoglucuronosyltransferase (UGT) after 15 min incubation, presented as a UGT positive control

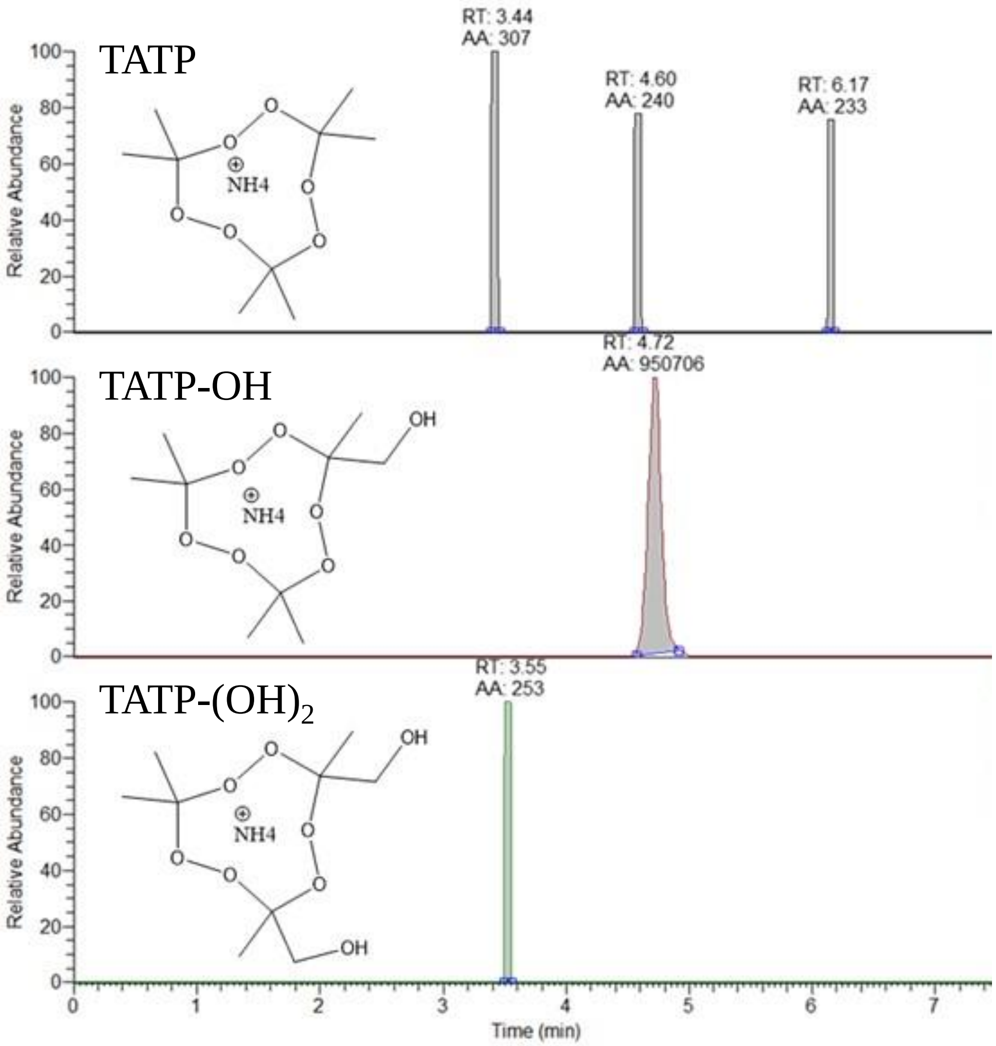

Fig. S4 Extracted ion chromatograms of triacetone triperoxide (TATP), hydroxy-triacetone triperoxide (TATP-OH) and dihydroxy-triacetone triperoxide (TATP-$(OH)_2$) from TATP-OH standard. No impurities were observed

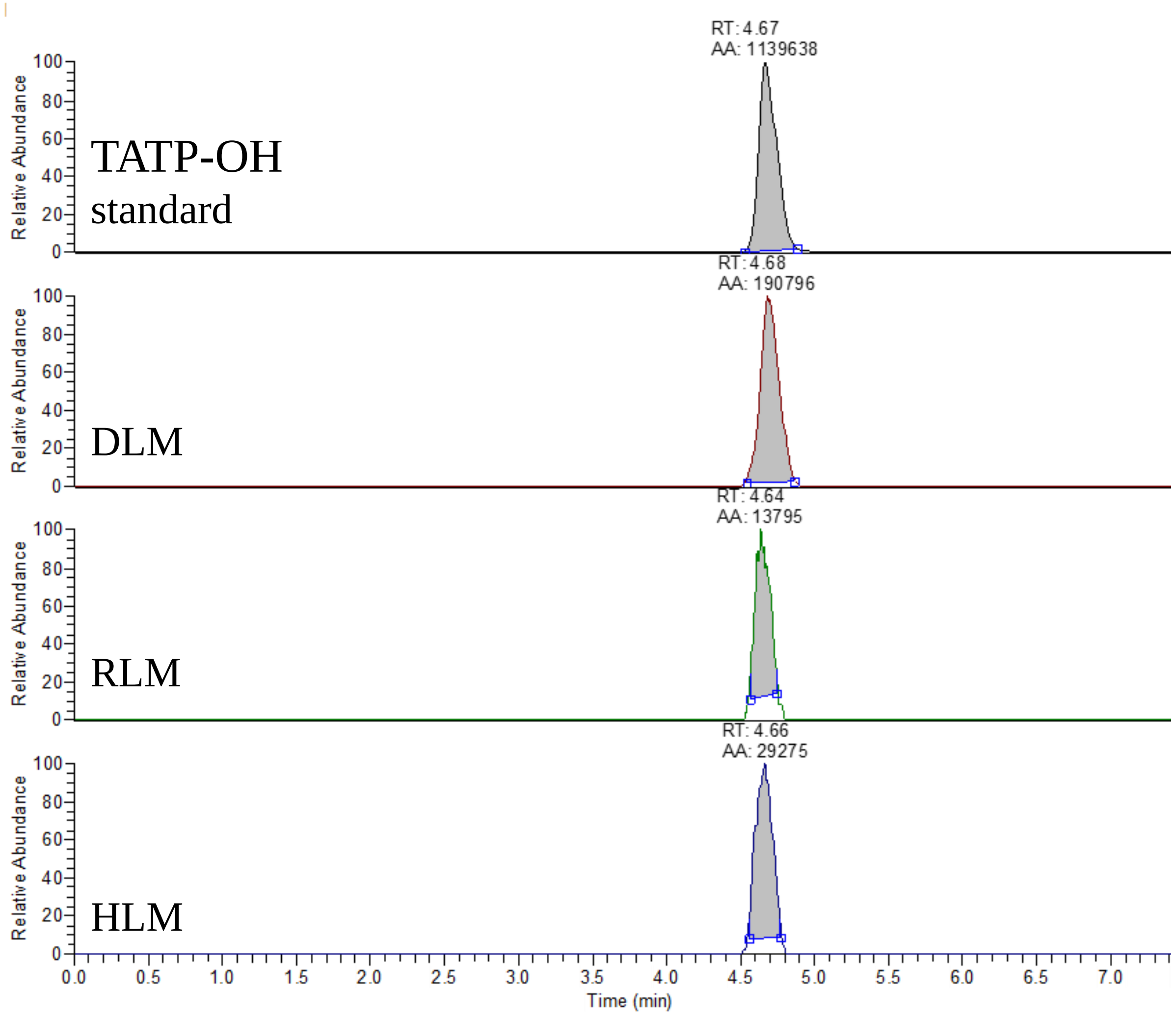

Fig. S5 Extracted ion chromatogram of TATP-OH from TATP incubations in human liver microsomes (HLM), dog liver mcirosomes (DLM) and rat liver microsomes (RLM). Different species exhibit the same metabolite, TATP-OH

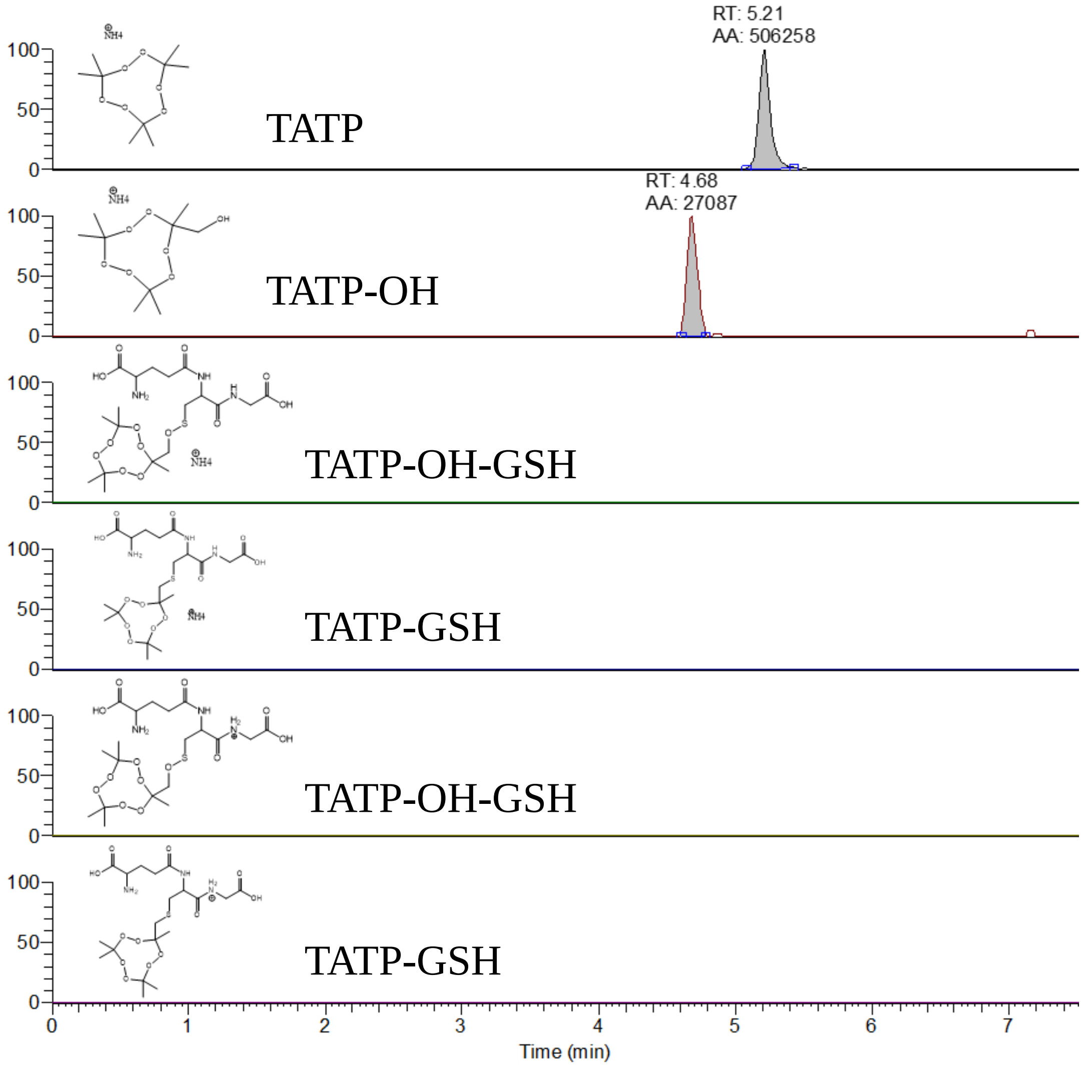

Fig. S6 Extracted ion chromatograms using atmospheric chemical ionization in positive mode (APCI+) of possible TATP reduced glutathione (GSH) metabolites after 2h incubation. $[M+H]^+$ and $[M+NH_4]^+$ are illustrated, though other adducts were searched for. Other ionization methods, such as electrospray ionization (ESI) in positive and negative mode and APCI in negative mode, were also tested (not shown). No TATP-GSH metabolites were identified.

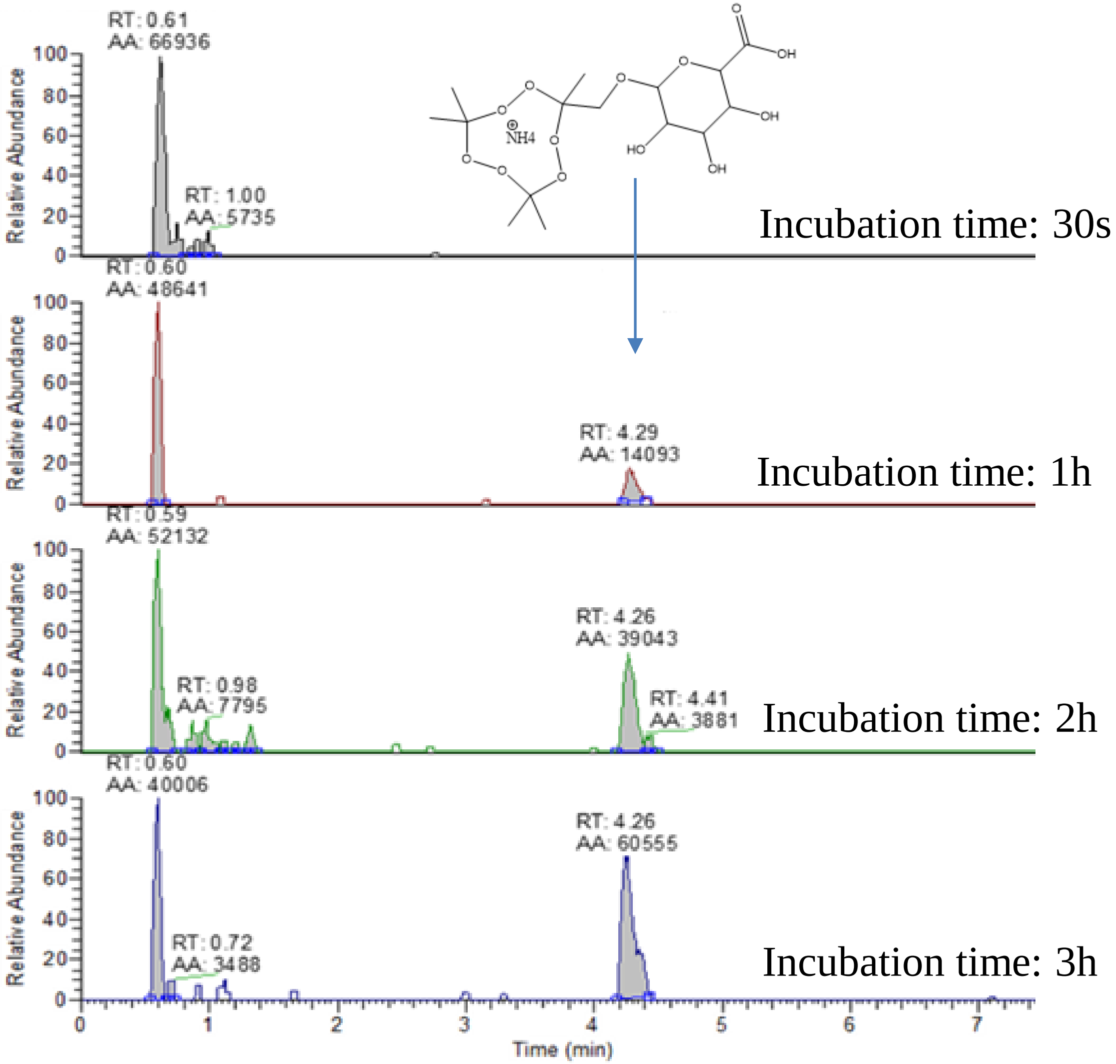

Fig. S7 Extracted ion chromatogram of [TATP-*O*-glucuronide + NH_4]$^+$ (*m/z* 432.1712) using APCI+, showing formation of TATP-*O*-glucuronide at 4.26 min as incubation of TATP progressed. Glucuronidation samples were concentrated prior to high-performance liquid chromatography–high-resolution mass spectrometry (HPLC–HRMS) analysis.

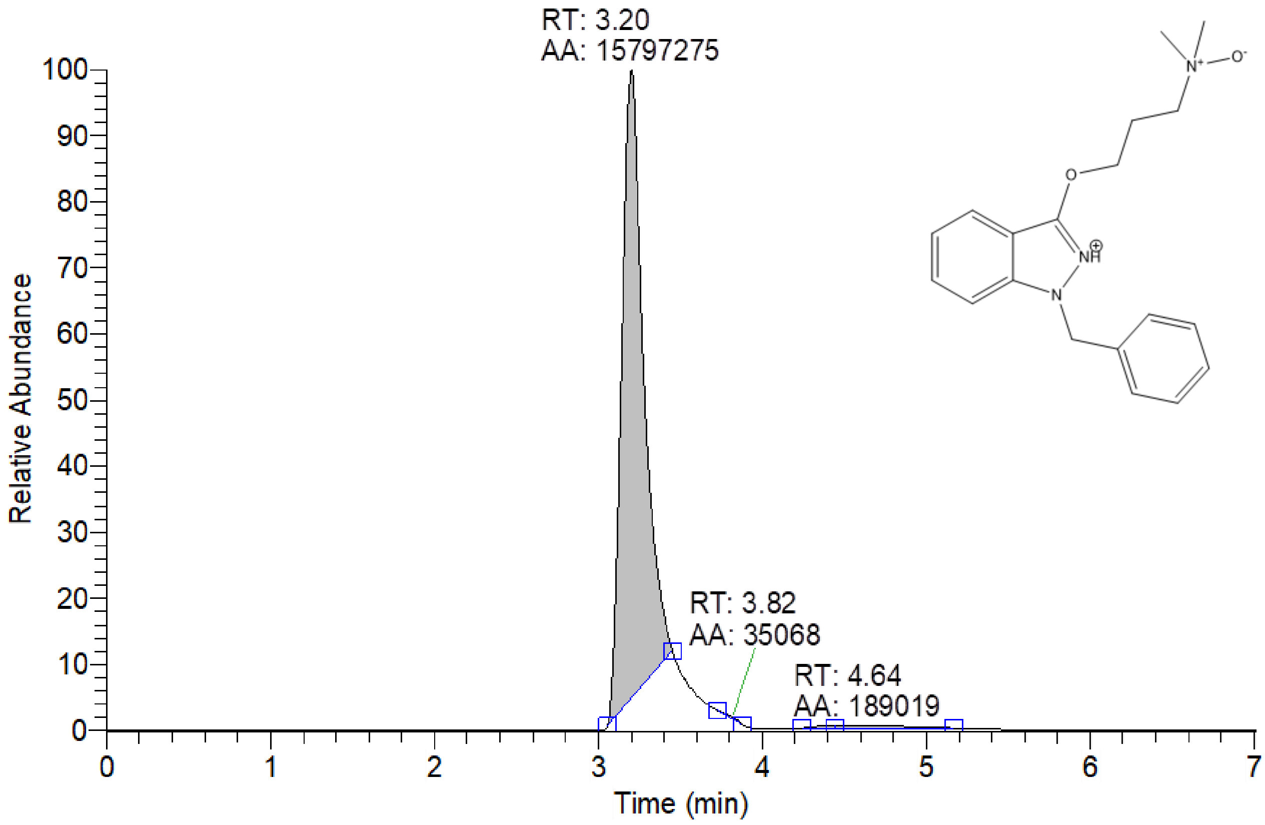

Fig. S8 Extracted ion chromatogram of benzydamine *N*-oxide metabolite from benzydamine metabolized by recombinant flavin monooxygenase 3 (rFMO3) after 10 min incubation, presented as a rFMO positive control

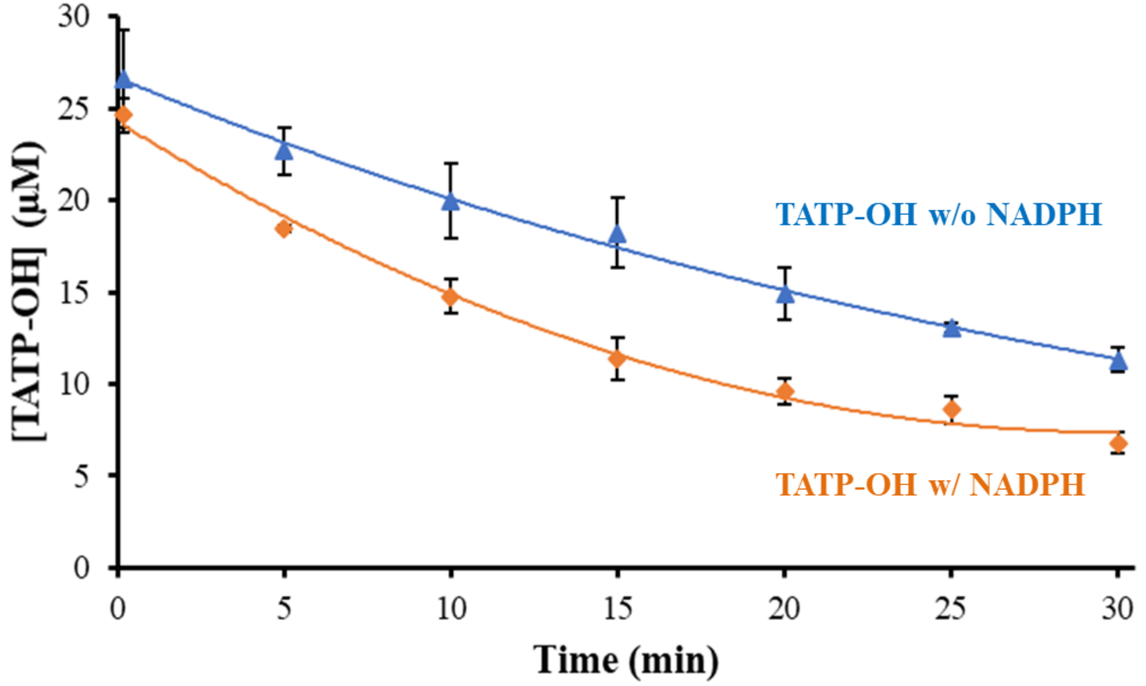

Fig. S9 TATP-OH depletion from incubations in CYP2B6 with (w/) or without (w/o) reduced nicotinamide adenine dinucleotide phosphate (NADPH). Performed in triplicates

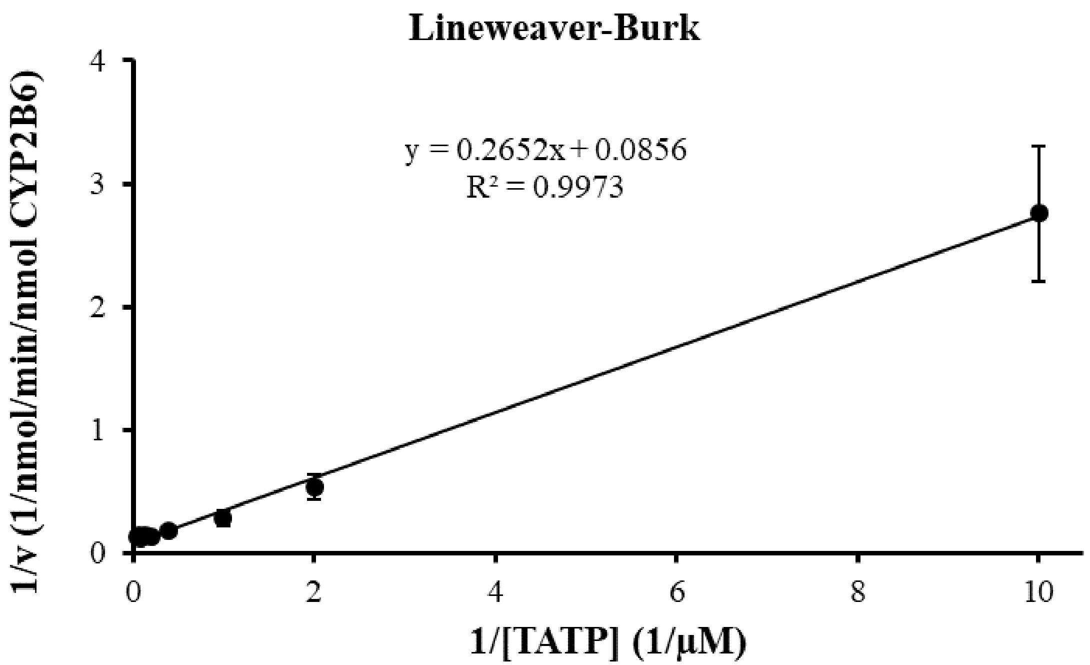

Fig. S10 Lineweaver-Burke plot, used to fit the equation: $1/v = (K_m/V_{max} \times 1/[S]) + 1/V_{max}$ to yield K_m = 3.1 µM and V_{max} = 11.7 nmol/min/nmol CYP2B6

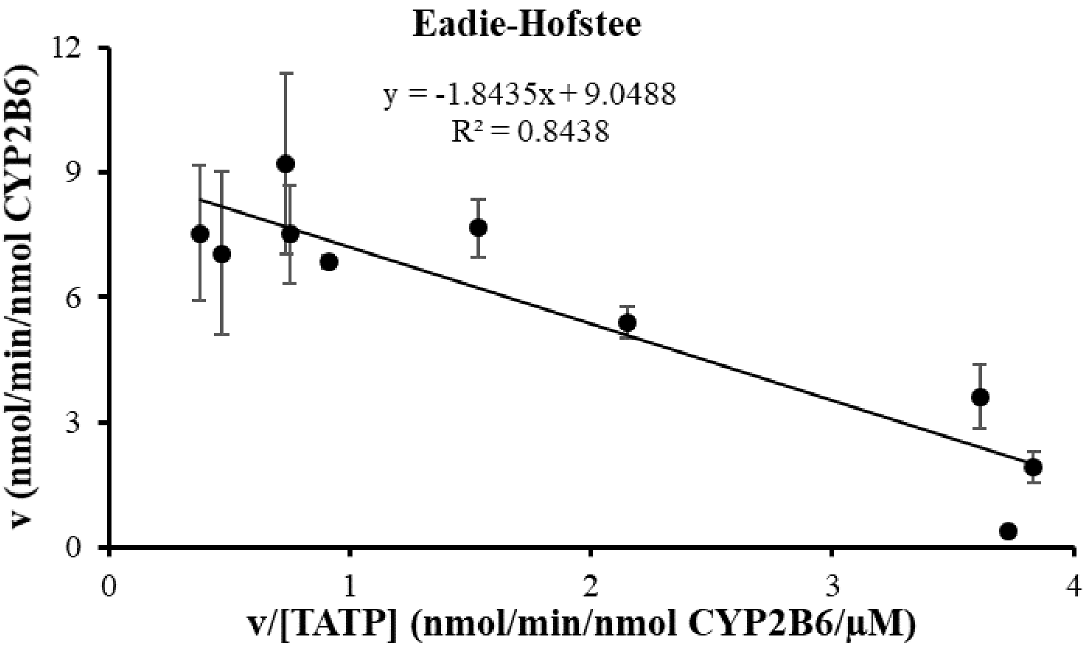

Fig. S11 Eadie-Hofstee plot, used to fit the equation: $v = (-K_m \times v/[S]) + V_{max}$ to yield K_m = 0.54 µM and V_{max} = 4.9 nmol/min/nmol CYP2B6

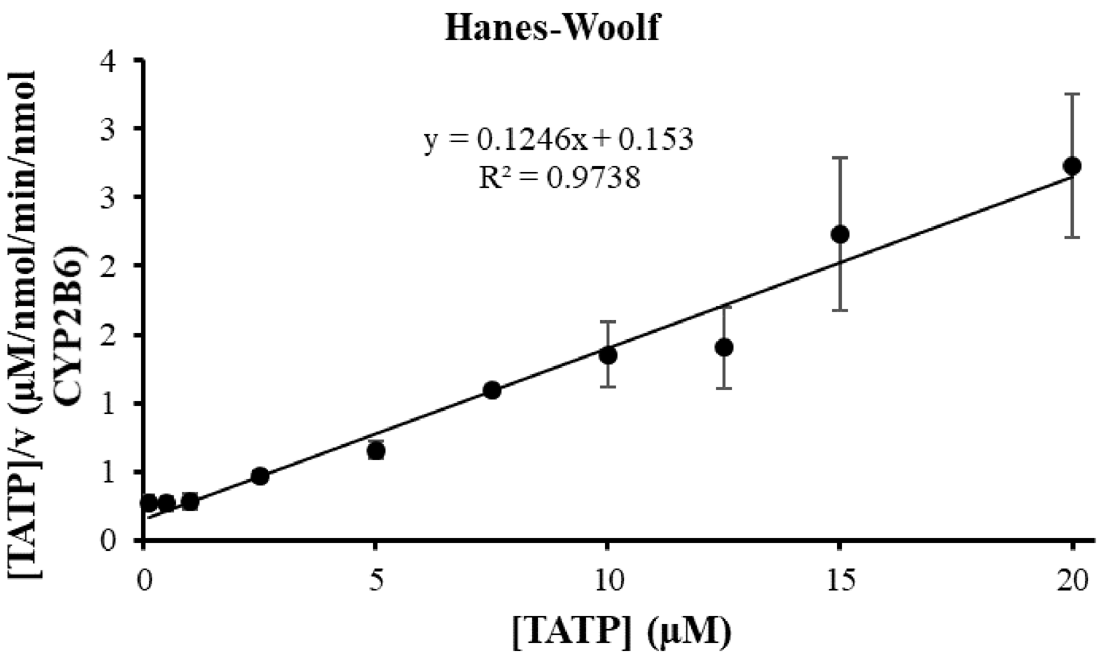

Fig. S12 Hanes-Woolf plot, used to fit the equation: $[S]/v = [S]/V_{max} + K_m/V_{max}$ to yield K_m = 1.2 µM and V_{max} = 8.0 nmol/min/nmol CYP2B6

Table S1 Hydroxy-triacetone triperoxide (TATP-OH) formation from triacetone triperoxide (TATP) incubations with recombinant cytochrome P450 (rCYP) and recombinant flavin monoxygenase (rFMO)

Incubation matrix	[TATP-OH] (μM)
HLM	1.47 ± 0.07
rCYP control	not observed
rCYP1A2	not observed
rCYP2B6	5.59 ± 0.3
rCYP2C9	not observed
rCYP2C19	not observed
rCYP2D6	not observed
rCYP2E1	not observed
rCYP3A4	not observed
rFMO control	not observed
rFMO1	not observed
rFMO3	not observed
rFMO5	not observed

Experiments with rCYP (100 pmol CYP/mL) or rFMO (100 μg protein/mL) consisted of 10 μg/mL TATP incubated with 10 mM phosphate buffer (pH 7.4), 2 mM $MgCl_2$ and 1 mM NADPH. Incubations were done in triplicates and quenched at 10 min

HLM human liver microsomes, *NADPH* reduced nicotinamide adenine dinucleotide phosphate

Table S2 TATP-*O*-glucuronide formation from TATP-OH incubations with recombinant uridine diphosphoglucuronosyltransferase (rUGT).

Incubation matrix	TATP-glucuronide/IS area count
HLM	0.26 ± 0.02
rUGT control	not observed
rUGT1A1	not observed
rUGT1A3	not observed
rUGT1A4	not observed
rUGT1A6	not observed
rUGT1A9	not observed
rUGT2B7	0.05 ± 0.03

Experiments with rUGT (500 µg protein/mL) consisted of 10 µg/mL TATP-OH incubated with 10 mM phosphate buffer (pH 7.4), 2 mM $MgCl_2$, 50 µg/mL alamethicin, 1 mM NADPH, and 5.5 mM UDPGA. Glucuronidation incubations were done in triplicates and quenched at 2 h. Quantification was done using area ratio TATP-*O*-glucuronide/IS. *UDPGA* uridine diphosphoglucuronic acid
